# Glutamine and amino acid metabolism as a prognostic signature and therapeutic target in endometrial cancer

**DOI:** 10.1002/cam4.6256

**Published:** 2023-06-30

**Authors:** Lirong Zhai, Xiao Yang, Yuan Cheng, Jianliu Wang

**Affiliations:** ^1^ Department of Obstetrics and Gynecology Peking University People's Hospital Beijing China

**Keywords:** amino acid, DNA repair, endometrial cancer, glutamine metabolism, immune response, nomogram, PHGDH, prognosis, serine metabolism

## Abstract

**Introduction:**

Endometrial cancer (EC) is the most common female reproductive system cancer in developed countries with growing incidence and associated mortality, which may be due to the growing prevalence of obesity. Metabolism reprogramming including glucose, amino acid, and lipid remodeling is a hallmark of tumors. Glutamine metabolism has been reported to participate in tumor proliferation and development. This study aimed to develop a glutamine metabolism‐related prognostic model for EC and explore potential targets for cancer treatment.

**Method:**

Transcriptomic data and survival outcome of EC were retrieved from The Cancer Genome Atlas (TCGA). Differentially expressed genes related to glutamine metabolism were recognized and utilized to build a prognostic model by univariate and multivariate Cox regressions. The model was confirmed in the training, testing, and the entire cohort. A nomogram combing prognostic model and clinicopathologic features was established and tested. Moreover, we explored the effect of a key metabolic enzyme, PHGDH, on the biological behavior of EC cell lines and xenograft model.

**Results:**

Five glutamine metabolism‐related genes, including PHGDH, OTC, ASRGL1, ASNS, and NR1H4, were involved in prognostic model construction. Kaplan–Meier curve suggested that patients recognized as high risk underwent inferior outcomes. The receiver operating characteristic (ROC) curve showed the model was sufficient to predict survival. Enrichment analysis recognized DNA replication and repair dysfunction in high‐risk patients whereas immune relevance analysis revealed low immune scores in the high‐risk group. Finally, a nomogram integrating the prognostic model and clinical factors was created and verified. Further, knockdown of PHGDH showed cell growth inhibition, increasing apoptosis, and reduced migration. Promisingly, NCT‐503, a PHGDH inhibitor, significantly repressed tumor growth in vivo (*p* = 0.0002).

**Conclusion:**

Our work established and validated a glutamine metabolism‐related prognostic model that favorably evaluates the prognosis of EC patients. DNA replication and repair may be the crucial point that linked glutamine metabolism, amino acid metabolism, and EC progression. High‐risk patients stratified by the model may not be sufficient for immune therapy. PHGDH might be a crucial target that links serine metabolism, glutamine metabolism as well as EC progression.

## Abstract 2

- The work is the first work to build a prognostic model regrading glutamine metabolism in endometrial cancer.
- The article found that DNA replication and repair dysfunction may be the key mechanism of glutamine metabolism reprogramming in EC progression.
- The article verified that one of the key genes, PHGDH, linking glutamine metabolism and serine metabolism, participates in EC progression and can be a promising therapeutic target using in vitro and in vivo experiments.

## 1 INTRODUCTION

EC is the most common gynecologic cancer in developed countries and unlike other cancers, the incidence and associated death of EC are both increasing annually. The growing prevalence of obesity mainly contributes to the rising incidence of EC and increasing cases in younger women. Although most patients are in an early stage can be controlled with surgery and adjuvant therapy with satisfied prognosis. However, patients with advanced and recurrent lesions lacking effective treatment undergo poor prognoses.^1^ Current approaches for evaluating prognosis are limited for considering clinicopathologic factors mainly. TCGA molecular classification has deepened our aspects of the molecular heterogeneity of EC. Thus, new approaches that take molecular biology and clinical factors into consideration for better prognosis prediction and precise treatment of EC are in need.

EC has the strongest relationship with obesity and metabolic disorders compared to other cancers. When body mass index (BMI) increases every 5 units, the risk of EC increases by more than 50%.^2^ The potential mechanism may involve an inflammatory environment, insulin signaling, and hyper estrogen transformation^3^; however, the exact mechanism of how metabolic disorder play a role in EC is still unclear. Our group has made several efforts to clarify the role of metabolic factors including glucose, insulin, and lipid on EC progression.^4^, ^5^, ^6^ Metabolic reprogramming plays a critical role in cancer growth and development.^7^ From Warburg's pioneer great work that identified glucose preference of cancer cells in the 1920s, tumor metabolic reprogramming has made much progress and is still a hot research topic full of unknowns today. In addition to glucose, glutamine is also an essential fuel and basic material for cancer energy consumption, macromolecular synthesis such as nucleotide,^8^ as well as immune response.^9^ Studies have revealed abnormal glutamine metabolism participates in the proliferation and development of hepatoma,^10^ lymphoma^11^ and cervix cancer.^12^ However, studies that explored the role of glutamine metabolism in EC are limited. Therefore, clarifying how glutamine metabolism functions in EC might provide a novel aspect to unveil the connection between metabolic disorder and EC, thus providing a novel target for EC treatment.

In recent years, bioinformatics as well as machine learning has developed as prevailing tools favoring diagnoses and treatments. Prognostic prediction tools like nomogram,^13^ a model that take clinicopathologic factors and molecular biology into consideration, can help stratify patients into different risk group and give precise treatments. Thus far, there has been no research that used genes related to glutamine metabolism to predict the prognosis of EC. Given the crucial function of glutamine in tumor existing and signaling, as well as the exact relevance of metabolic dysfunction and EC, we assumed that a model based on glutamine metabolism would be worthy for EC prognosis evaluation. In this study, we identified five glutamine metabolism‐related genes as independent prognostic factors in EC. We then built a prognostic nomogram integrating five key genes and clinical factors that has good prediction ability. The present model focused on the glutamine metabolism or the genes that interacted with glutamine regulation, which is more specific than our previous work which focuses on the whole metabolism including glucose, lipid, and amino acid.^14^ The work may give new insights into the mechanism of how glutamine metabolism regulation network participates in EC progression and provide new target for cancer therapy. One of the five key genes, PHGDH, which may be an important node linking serine metabolism and glutamine metabolism, was verified in vitro and in vivo, thus may provide a promising target for EC treatment.

## 2 METHODS AND MATERIALS

### 2.1 Data acquisition and preparation

The mRNA expression data and clinical profiles of 552 ECs and 35 normal samples were downloaded from TCGA (https://portal.gdc.cancer.gov/). Patients with reachable mRNA expression data and entire clinical profiles including age, histology, lymph node metastasis (LNM), stage, grade, peritoneal cytology, and prognostic data like survival status and time were included in the following analyses.

### 2.2 Identification of differentially expressed glutamine metabolism‐related genes in EC

Glutamine metabolism‐related gene sets (REACTOME_GLUTAMATE_AND_GLUTAMINE_METABOLISM.v7.5.1; GOBP_REGULATION_OF_GLUTAMINE_FAMILY_AMINO_ACID_METABOLIC_PROCESS.v7.5.1; GOBP_GLUTAMINE_FAMILY_AMINO_ACID_METABOLIC_PROCESS.v7.5.1; GOBP_GLUTAMINE_FAMILY_AMINO_ACID_CATABOLIC_PROCESS.v7.5.1; GOBP_GLUTAMINE_FAMILY_AMINO_ACID_BIOSYNTHETIC_PROCESS.v7.5.1; GOBP_PEPTIDYL_GLUTAMINE_METHYLATION.v7.5.1) were obtained from the Molecular Signatures Database, v5.1 (MSigDB) (http://www.broad.mit.edu/gsea/msigdb/). Gene Ontology (GO) and Kyoto Encyclopedia of Genes and Genomes (KEGG) pathway enrichment analyses were performed to analyze the enriched metabolism and signaling pathways of these glutamine metabolism‐related genes using ClusterProfiler R package, and adjusted *p* values <0.05 were considered to indicate significant statistical significance.

We extracted glutamine metabolism‐related gene expression data from above‐mentioned mRNA profiles. Then, the glutamine metabolism‐related genes that were differentially expressed between EC and normal samples were screened out using the limma R package and Wilcox test, with a false discovery rate (FDR) < 0.05 and an absolute log2‐fold change (|logFC|) > 1 as the cut‐off values. R × 64 3.6.3 software was used for data analysis.

### 2.3 Construction and verification of glutamine metabolism‐related gene prognostic model

We initially used the “survival” R package to figure out genes extracted from 2.2 that were also closely related to the survival of EC patients given by univariate Cox regression analysis. Genes with statistical significance were entered into the subsequent analysis. The entire cohort (*n* = 542, with complete survival material) was separated into the training (*n* = 272) and testing cohort (*n* = 270) randomly. A multivariate Cox regression was completed based on the training cohort to shape a prognostic model. The risk score of individual patients can be estimated by the prognostic index formula, as follows^15^:

$$\text{Risk score}=\sum n_{i}=\sum \left(\text{Coefi}*\mathrm{Exp}x_{i}\right).$$

Coefi denotes the coefficient of individual gene in the model, while Expx_i_ signifies the expression level of each gene.

Patients in the entire and separated cohort were divided into corresponding risk groups cut by the median risk score calculated by the prognostic model. Kaplan–Meier survival curve and time‐dependent ROC analysis were performed to examine the prediction efficiency of the model to estimate prognosis by the R packages named “survminer,” “survival,” and “survival ROC,” with an area under the curve (AUC) beyond 0.60 considered suitable.

We use the “beeswarm” R package to identify the association between the prognostic model and clinicopathologic factors. Patients with different clinicopathologic features were split into high‐ and low‐risk groups to analyze whether the model was independent to predict prognosis.

### 2.4 Proportional hazard assumption test

In this study, we used Cox regression analysis to construct a nomogram. Meeting the proportional hazard assumption (PHA) is a prerequisite for applying the Cox model.^16^, ^17^ If the *p* value >0.05 indicates that the null hypothesis, which is the covariate satisfies the proportional hazard hypothesis, is not rejected and the risk of this covariate is a time constant, the Cox model can be used to study. We performed this test using the “survival” R package based on the Schoenfeld residuals method. The horizontal axis on the residual plot represents time, and if the residuals are evenly distributed, it means that the residuals are independent of time.

### 2.5 Construction and validation of a nomogram based on the risk signature

Univariate and multivariate Cox regressions of the glutamine metabolism‐based risk score and several clinicopathologic factors were used to identify whether the present model has an independent prognostic value. A nomogram^18^ was established using the “regplot” R package based on the risk score and clinicopathologic features to simplify risk estimation and prognosis prediction of EC patients. The accuracy of the nomogram was analyzed by the calibration curve.

### 2.6 Enrichment analysis of the intersection genes

GSEA^19^ was accomplished to identify the potential signaling pathways that participate in the regulation of different risk groups graded by the prognostic model. The gene set was selected by the FDR *q* value ≤0.25, absolute normalized enrichment score (|NES|) ≥1.0, and nominal (NOM) *p* value ≤0.05.

### 2.7 Relevance of the glutamine metabolism‐related prognostic model with tumor immune microenvironment

The tumor immune environment consists of immune cells and stromal cells. We used the “estimate” package in R software to calculate stromal and immune cell scores for 542 samples. The estimation score is the combination of the stromal score and immune score. We analyzed these scores in different risk groups based on our glutamine metabolism‐related gene prognostic model by *t*‐test. A lower score means a lower occurrence of the corresponding component.

### 2.8 Correlation of genes in the prognostic model with clinicopathologic features and interacting network analysis

We analyzed the correlation of five key genes concluded in the prognostic model with clinical features (LNM, stage, peritoneal cytology, grade, tumor state, and survival status) of EC patients by the Wilcoxon test. *p* < 0.05 was recognized as statistically significant. GeneMANIA (http://genemania.org/) was used to analyze genes that were interacted with prognostic model genes.

### 2.9 Bioinformatic analysis of PHGDH

Survival analysis based on the protein expression of PHGDH on EC and normal samples was retrieved from Human Protein Atlas database (HPA) (https://www.proteinatlas.org/). GSEA was used to perform single gene enrichment analysis of PHGDH.

### 2.10 Immunohistochemistry

The tissues of EC and para‐carcinoma are from patients treated at the Department of Obstetrics and Gynecology of Peking University People's Hospital (PKUPH) (Beijing, China). The informed consents have been obtained from the patients. The study was approved by the Ethics Committee of the People's Hospital, Peking University (2020PHB424‐01). The immunohistochemistry (IHC) was performed as previously described^20^ with anti‐PHGDH (Proteintech, 14719‐1‐AP). Percentage of the positive cells were analyzed by ImageJ software (Rawak Software, Inc. Germany). Statistical analysis was performed using Student's *t*‐test by GraphPad Prism 8 (GraphPad Software Inc., La Jolla, CA, USA). The *p* values were provided.

### 2.11 Cell culture and materials

EC cell lines Ishikawa, AN3CA, HEC50B, RL952, HEC1B, KLE, and HEC1A were obtained from the American Type Culture Collection (Manassas, VA, USA) and maintained in our laboratory. The cell lines were cultured in DMED/F12 or MEM medium with 10% or 15% FBS according to the provider's instructions and incubated at 37°C with 5% CO2. PHGDH inhibitor, NCT‐503 (S8619) was purchased from Selleck. siRNAs targeting PHGDH (si1‐PHGDH: ss 5′‐CGGAUGUGAACUUGGUGAATT‐3′, as 5′‐UUCACCAAGUUCACAUCCGTT‐3′; si2‐PHGDH: ss 5′‐GCAGAACUCACUUGUGGAATT‐3′, as 5′‐UUCCACAAGUGAGUUCUGCTT‐3′; si3‐PHGDH: ss 5′‐CGACAGGCUUGCUGAAUGATT‐3′, as 5′‐UCAUUCAGCAAGCCUGUCGTT‐3′.) were purchased from Tsingke Co. Ltd (Beijing, China).

### 2.12 Transfection and validation

Cells were transfected with siRNA using Lipofectamine 3000 reagent (Invitrogen Life Technologies, Carlsbad, CA, USA) along with the manufacturer's procedure within 24 h after seeded in the 6‐well culture plates. RNA and total protein were extracted 48 or 72 h after transfection for further analysis. The efficiency of transfection was validated by western blot and real‐time qPCR. Western blot was conducted as previously described.^21^ Antibodies used in the present study were: anti‐PHGDH (Proteintech, 14719‐1‐AP), anti‐β‐actin (Proteintech, 60008‐1‐Ig), anti E‐cadherin (Proteintech, 20874‐1‐AP), anti‐vimentin (Proteintech, 60330‐1‐Ig), anti‐caspase‐3 (Proteintech, 66470‐2‐Ig), anti‐Zeb1 (CST, 70512T), anti‐β‐catenin (CST, #9562), anti‐N‐cadherin (abcam, ab76011), anti‐Snail (CST, 3879T), anti‐caspase‐9 (Proteintech, 66169‐1‐Ig), anti‐Bax (Proteintech, 50599‐2‐Ig), and anti‐Bcl‐2 (Proteintech, 60178‐1‐Ig). Real‐time qPCR was conducted along with the manufacturer's protocols. Primers used were PHGDH (Forward: GAATGATCATGTGCCTGGC; Reverse: GTTCCCATGAACTTCTTCCG).

### 2.13 Cell proliferation assay

The cell viability and proliferation were evidenced by Cell Counting Kit (CCK‐8) (Dojindo, Japan) at 450 nm using a microplate reader. For analysis of apoptosis rate, cells were double stained with FITC‐annexin V and propidium iodide (PI) according to the Annexin V/FITC apoptosis assay kit (BD, USA) and tested by flow cytometer (BD, USA). All of the experiments were repeated three times independently. The statistical analysis was performed using Student's *t*‐test by GraphPad Prism 8 (GraphPad Software Inc., La Jolla, CA, USA). The *p* values were provided.

### 2.14 Cell migration assay

The effect of PHGDH on the metastases of EC cells was evaluated by the wound healing and transwell migration assay. Cells seeded in the 6‐well plate were cultured into an 85% density monolayer. Then the monolayer cell on the plates was scratched with 200 μL sterile pipette tips. The cell debris was washed away with PBS. The wound was imaged every 24 h. For transwell assay, the 100 μL serum‐free medium containing 20,000 cells was added to the upper chambers, while 500 μL medium containing 10% FBS was added to the lower chambers, and then incubated for 48 h. Cancer cells were fixed with 4% paraformaldehyde for 30 min, stained with 0.1% crystal violet for 5 min, washed three times with PBS, and counted in six fields under the microscope. Images were statistically analyzed using ImageJ software (Rawak Software, Inc. Germany). All of the experiments were repeated three times independently. The statistical analysis was performed using Student's *t*‐test by GraphPad Prism 8 (GraphPad Software Inc., La Jolla, CA, USA). The *p* values were provided.

### 2.15 Animal experiment

All animal care and procedures were in accordance with national and institutional policies for animal health and well‐being and approved by the Laboratory Animal Ethics Committee of Peking University People Hospital (Ethics approval number: 2020PHE094). Female BALB/c nude mice aged 5 weeks were purchased from Vital River Laboratory Animal Technology Co. Ltd (Beijing, China). After adaptation about 1 week, the mice were subcutaneously injected with 100 μl serum‐free medium containing 3 × 10^6^ Ishikawa cells. The tumor volume [(major axis) × (minor axis)2 × 1/2] and body weight of each mouse were documented every 3 days. The mice were randomly allocated into two groups (*n* = 6 per group) when tumor volume reached 50 mm^3^, one group received intraperitoneal injection of NCT‐503^22^ (20 mg/kg) every 2 days; the control group was given an equal volume of solvent. When the tumor volume reached 2 cm^3^, mice were sacrificed to measure the tumor weight and tumor volume.

### 2.16 Statistical method

Continuous variables were summarized as mean ($\bar{X}$) ± SD or median; categorical variables were described by frequency (*n*) and proportion (%). Differences among variables were tested using Student's *t*‐tests (two groups) or one‐way ANOVA tests (above two groups). The log‐rank test was applied to compare the OS rates of the high‐risk and low‐risk groups. Univaribale and multivariable logistic COX regression analyses were applied to calculate the hazard ratio (HR) and its 95% confidence interval (CI). Statistical analyses were performed using R software (version×64 3.6.3) or GraphPad Prism 8 (GraphPad Software Inc., La Jolla, CA, USA). All statistical tests were two‐sided, and a *p*‐value of 0.05 was used to indicate statistical significance.

## 3 RESULTS

### 3.1 Identification of glutamine metabolism‐related differentially expressed genes

The workflow of the present study is represented in Figure S1.

**FIGURE 1:**
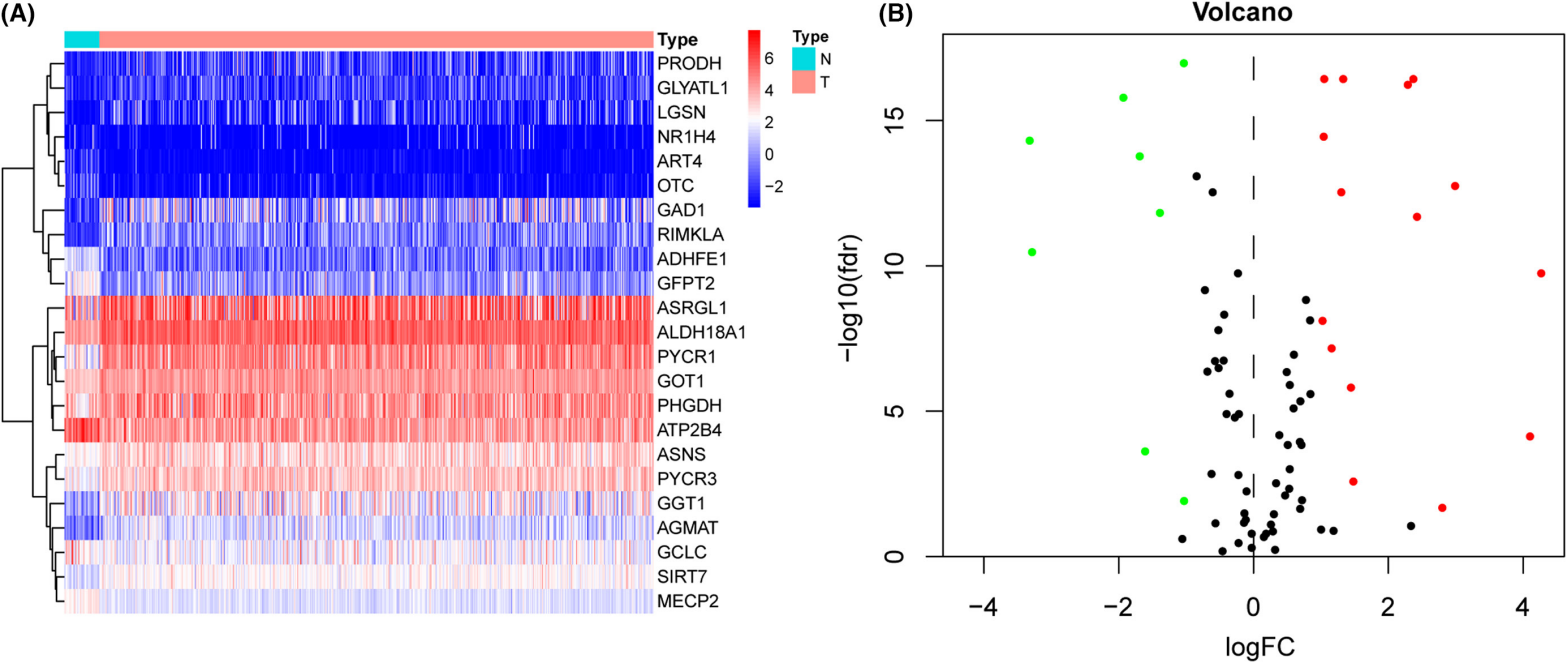
Identification of differently expressed glutamine metabolism‐related genes in EC. The heatmap (A) and volcano plot (B) for DEGs where red and green dots represent upregulated and downregulated genes.

A cohort of 552 endometrial cancer and 35 normal tissue samples was extracted from TCGA with their clinicopathologic characteristics. We downloaded and reviewed 152 glutamine metabolism‐related genes from MSigDB and extracted their expression data from TCGA. Enrichment analysis showed that these glutamine metabolism‐related genes also participated in other amino acid metabolism such as alanine, aspartate, arginine, cystenine, and methione metabolism, indicating their intertwined regulation network (Figure S2). Twenty‐three differentially expressed glutamine metabolism‐related genes were identified (FDR <0.05, |logFC| > 1) as shown in the heatmap and the volcano plot (Table S1, Figure 1A,B). Of these 23 DEGs, 15 were upregulated and 8 were downregulated.

### 3.2 Prognostic model establishment based on differentially expressed genes

**FIGURE 2:**
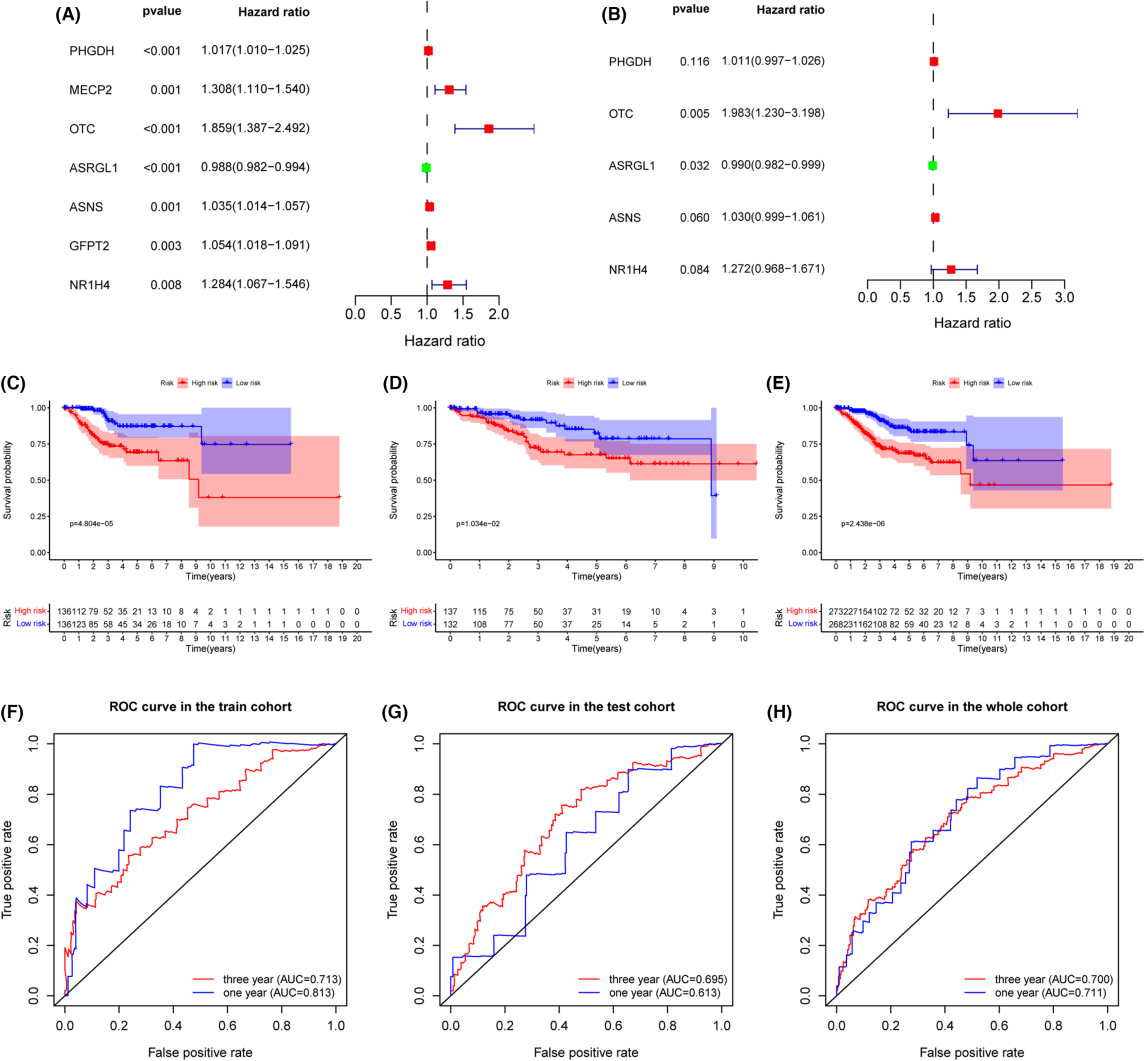
Univariate and multivariate Cox regressions to recognize independent prognostic glutamine metabolism‐related genes of EC in the training cohort. Kaplan–Meier survival and ROC curve were performed to validate the predicting efficacy of the prognostic signature. (A) Univariate Cox analysis. Hazard ratio (HR) >1 indicates that exposure is a risk factor, and HR <1 indicates that exposure is a protective factor. (B) Multivariate Cox analysis. (C–E) Kaplan–Meier survival for EC patients of high‐ and low‐risk groups in the training cohort, testing cohort, and the entire cohort, respectively. (F–H) ROC curves based on the prognostic signature in the training cohort, testing cohort, and the entire cohort respectively.

Univariate Cox regression was conducted to screen out overall‐survival associated glutamine metabolism‐related genes. Seven genes were finally obtained for further research (*p* < 0.01) (Figure 2A). To further screen for independent prognostic genes that affect patient survival, we further divided all patients into the Training (*n* = 272) and Testing (*n* = 270) sets. Ultimately, the multivariate Cox regression showed that there were five genes including PHGDH, OTC, ASRGL1, ASNS, and NR1H4 incorporated into the risk score and the risk score was an independent prognostic factor affecting overall survival in the training cohort. Then, we established a prognostic model based on the five genes (Figure 2B, Table 1), and the risk score was calculated as follows:

$$\begin{array}{cc} \text{Risk score}\; & =\left(0.01124^{*}\;\text{PHGDH}\right)\\ & +\left(0.684812436742728^{*}\;\mathrm{OTC}\right)\\ & +\left(-0.00972165906799014^{*}\;\text{ASRGL1}\right)\\ & +\left(0.0292234328730247^{*}\;\text{ASNS}\right)\\ & +\left(0.240358445815512^{*}\;\mathrm{NR}1\text{H4}\right) \end{array}$$

**TABLE 1:** The multivariate regression coefficients of each gene.

ID	Coef	HR	CI	*p* Value
PHGDH	0.011239	1.011302	(0.997216, 1.025588)	0.11633
OTC	0.684812	1.9834	(1.23023, 3.197675)	0.00495
ASRGL1	−0.00972	0.990325	(0.981564, 0.999165)	0.032017
ASNS	0.029223	1.029655	(0.998813, 1.061449)	0.059643
NR1H4	0.240358	1.271705	(0.967835, 1.67098)	0.084475

Abbreviations: CI, confidence interval of HR; Coef, coefficient; HR, hazard ratio.

**FIGURE 3:**
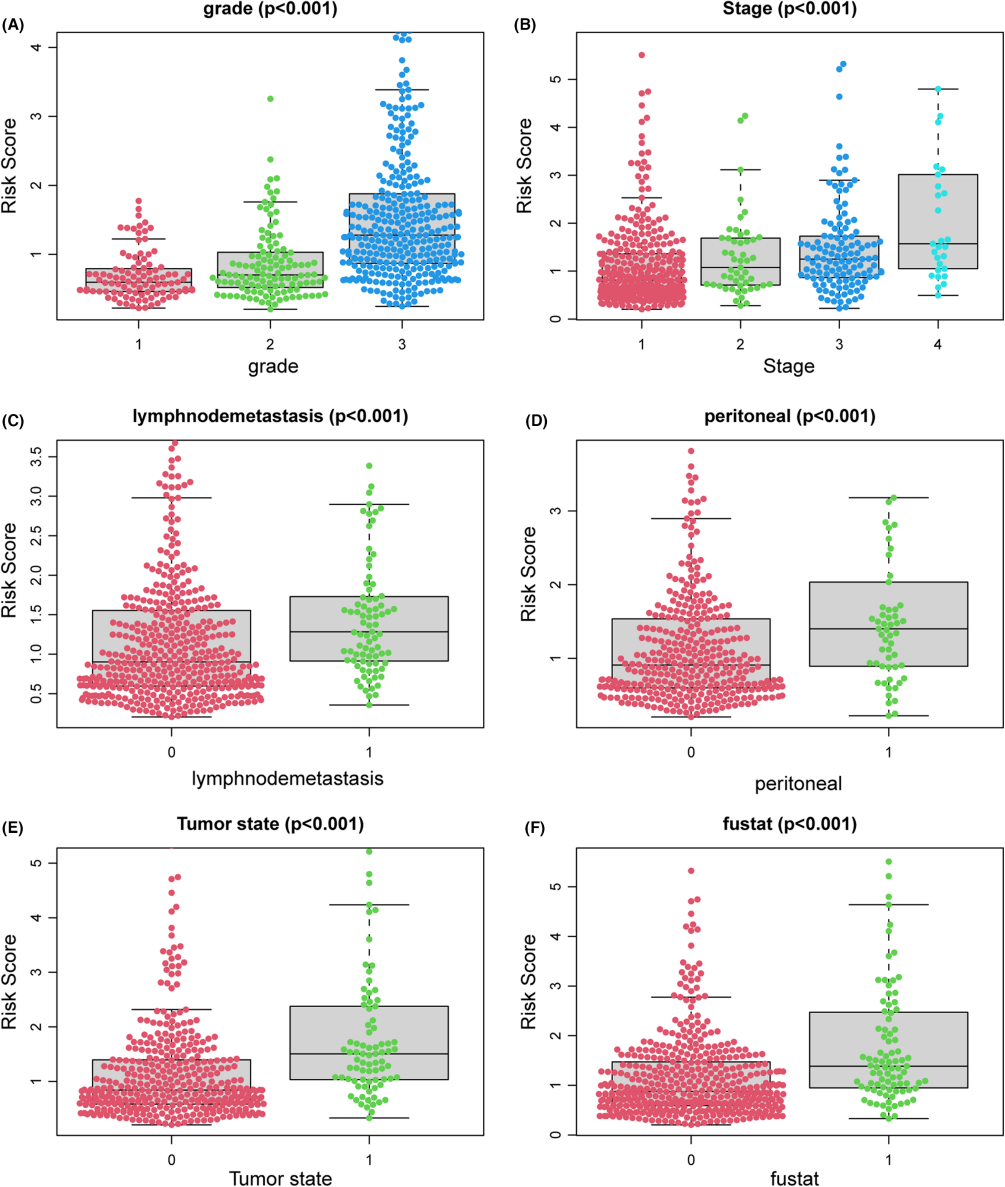
Association between the glutamine metabolism‐related risk signature and clinicopathologic features. (A) Grade. (B) Stage. (C) Lymphnode metastasis. (D) Peritoneal cytology. (E) Tumor status. (F) Status of dead.

**FIGURE 4:**
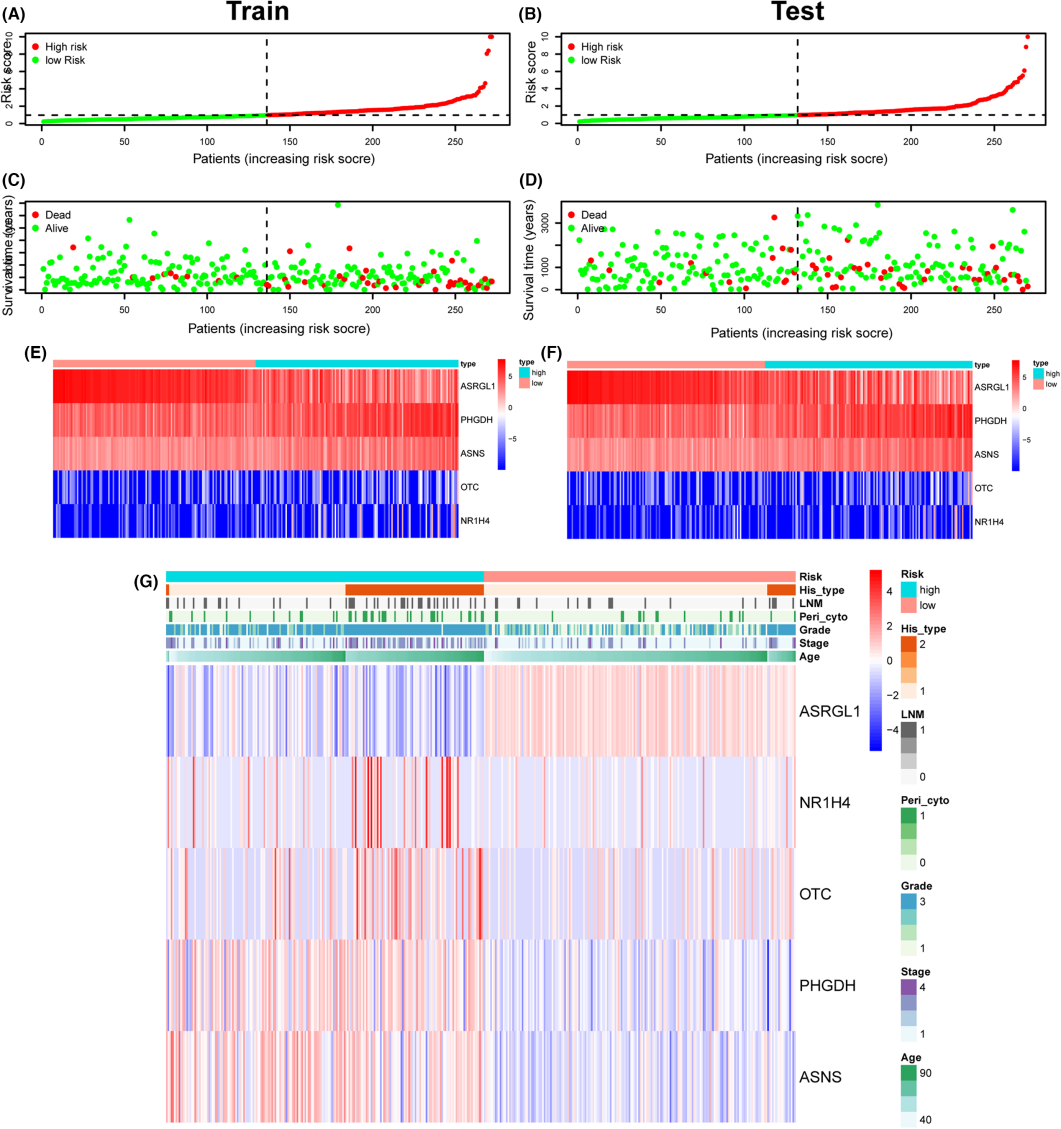
Glutamine metabolism‐related risk signature. Risk curve (A), survival status (C) and prognosis model gene expression (E) were displayed according to high‐ and low‐risk groups in the training cohort. Risk curve (B), survival status (D), and prognosis model gene expression (F) were displayed according to high‐ and low‐risk groups in the testing cohorts. (G) The heatmap displayed risk gene expression in the prognostic model according to different risk, survival status, and clinicopathologic characteristics in the entire cohort.

To further validate the prediction accuracy of this prognostic model, we subdivided patients in the training set into high‐ and low‐risk groups along with the median risk score. The efficacy of the prognostic model for predicting prognosis was estimated by the Kaplan–Meier survival and ROC curve. Survival analysis indicated that patients with high risk have a worse prognosis than patients with low risk in the training cohort (Figure 2C), which was consistent with the results in testing and the entire cohort when dividing patients into two risk groups accordingly, suggesting the predictive ability of the prognostic model was sufficient (Figure 2D,E). The ROC curve verified an acceptable accuracy of the prognostic model for predicting 1‐year (AUC = 0.813, 0.613, and 0.711) and 3‐year (AUC = 0.713, 0.695, and 0.700) prognosis whether in the training, testing, or entire cohort (Figure 2F–H). In addition, the risk score was shown to be related to the clinicopathologic characteristics including grade, stage, peritoneal cytology, lymph node metastasis, tumor state, and status of the dead (Figure 3A–F). The survival status and expression of five genes in the training and testing set were visualized according to the risk curve. Patients in the high‐risk group revealed more deaths as expected (Figure 4A–F). The heatmap revealed the mRNA expression of five key genes along with risk levels, survival status, and clinicopathologic features of EC patients (Figure 4G). Taken together, these results showed that the model based on these five hub genes had a good predictive ability to evaluate the survival of EC patients.

**FIGURE 5:**
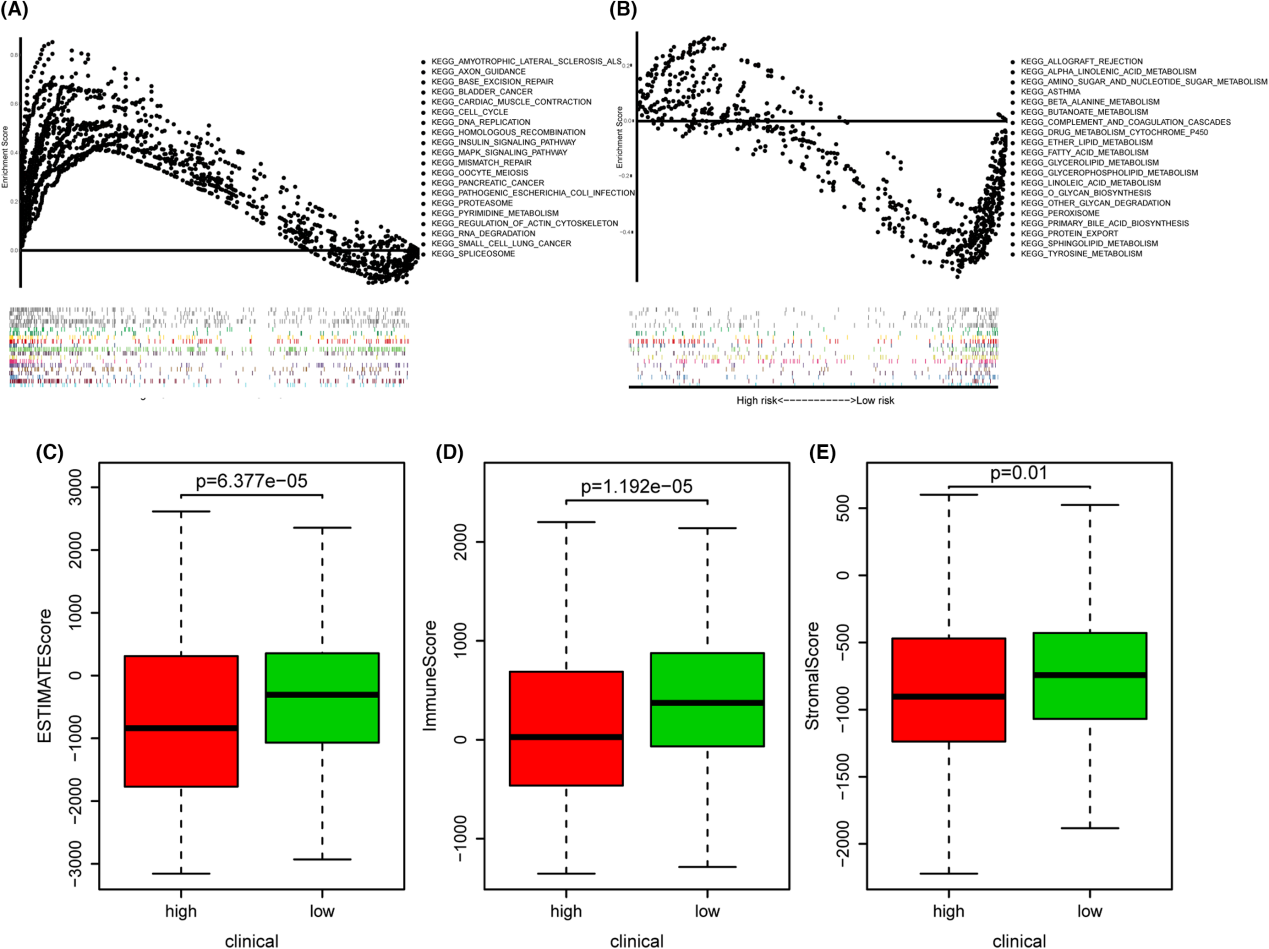
Cancer signaling pathways and tumor immune environment in different risk groups stratified by glutamine metabolism‐related prognostic model. The GSEA analysis of DEGs between two risk groups. KEGG enrichment of high‐risk group (A) and low‐risk group (B). The distribution of (C) estimation scores, (D) immune scores, and (E) stromal scores in high‐ and low‐risk groups.

In addition, to further reveal the signaling pathways in which this prognostic model participates, we performed the KEGG pathway analysis of the two risk groups by GSEA. The high‐risk group showed higher cell cycle, homologous recombination. DNA replication, insulin signaling, MAPK pathway, and mismatch repair (Figure 5A), whereas the low‐risk group exhibited higher tyrosine metabolism and sphingolipid metabolism (Figure 5B). Nevertheless, the glutamine metabolism‐related signature‐based risk group might be valuable for distinguishing high‐ and low‐risk EC patients and the underlying mechanism indicated to DNA repair, insulin signaling pathway, and metabolism network.

Next, we explored whether the risk signature could identify patients with good immune responses. The result suggested that high‐risk patients stratified by our glutamine metabolism‐related prognostic model was significantly related to lower estimation score (*p* < 0.001), immune score (*p* < 0.001), as well as stromal score (*p* = 0.01) (Figure 5C–E), indicating an inferior immune response that may not be suitable for immune therapy.

### 3.3 Building a prognostic nomogram for EC patients

**FIGURE 6:**
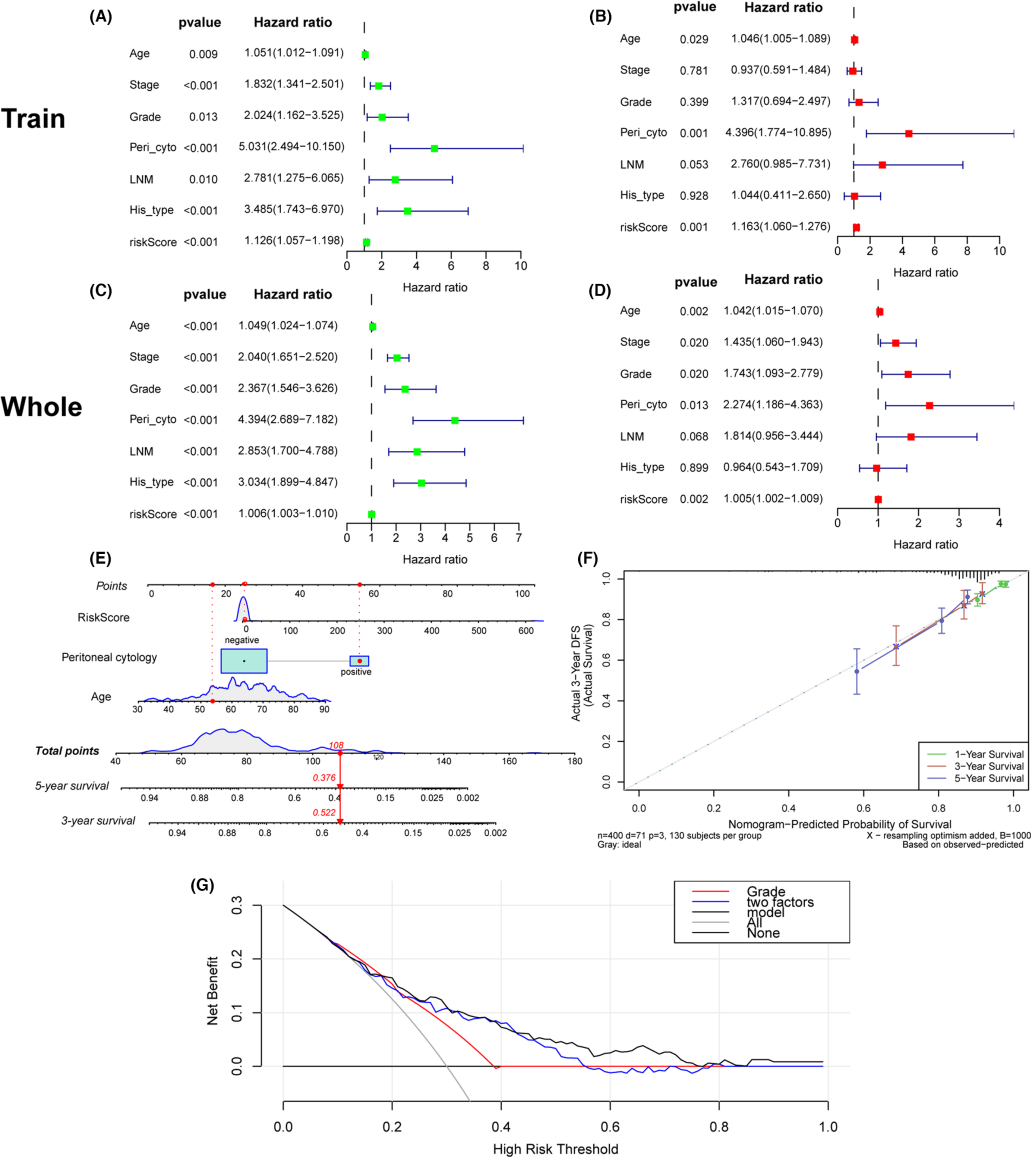
Univariate and multivariate Cox regression analyses of overall survival in EC patients. Univariate analysis of the traning cohort (A) and the entire cohort (C). Multivariate analysis of the traning cohort (B) and the entire cohort (D). (E) Nomogram for predicting the probability of 1‐, 3‐, and 5‐year OS for the prognosis of EC patients. Three factors were included in this nomogram. (F) Calibration plot of the nomogram for predicting the probability of OS at 1, 3, and 5 years. (G) The DCA curve indicated that the prediction ability of the nomogram based on these three factors was great than any one or two factors.

To explore whether the glutamine metabolism‐related risk score is an independent prognostic factor, we conducted univariate and multivariate Cox regressions in the training cohort. The univariate analysis reported that age, stage, grade, histological type, peritoneal cytology, LNM, as well as the risk score were significant prognostic factors (*p* < 0.01) (Figure 6A,C). Multivariate Cox analysis showed that risk score was an independent factor affecting the prognosis of EC patients (Figure 6B,D) (*p* < 0.001). In addition, peritoneal cytology and age were also shown to be independent for EC prognosis in the training cohort (Figure 6B,D).

We performed the PHA test on all variables included in the univariate and multivariate Cox regression analyses. Figure S3 shows that in the Schoenfeld residual curve, all variables' residuals were evenly distributed which indicates that these variables do not change over time. All variables and the entire model satisfied the PHA hypothesis (every covariate *p* > 0.05 and global *p* > 0.05).

To better evaluate the risk stratification and prognosis, we construct a nomogram with risk score and other two independent prognostic features including age and peritoneal cytology identified in the training cohort (Figure 6E). The nomogram makes it convenient for clinical application by predicting survival with a line linking total scores and survival rate. Besides, the calibration curve indicated the accuracy of our nomogram as the predicted survival rate of 1, 3, and 5 years was near to the actual result (Figure 6F). The DCA analysis showed better predictive performance of our nomogram based on these three factors than any one or two features (Figure 6G).

**FIGURE 7:**
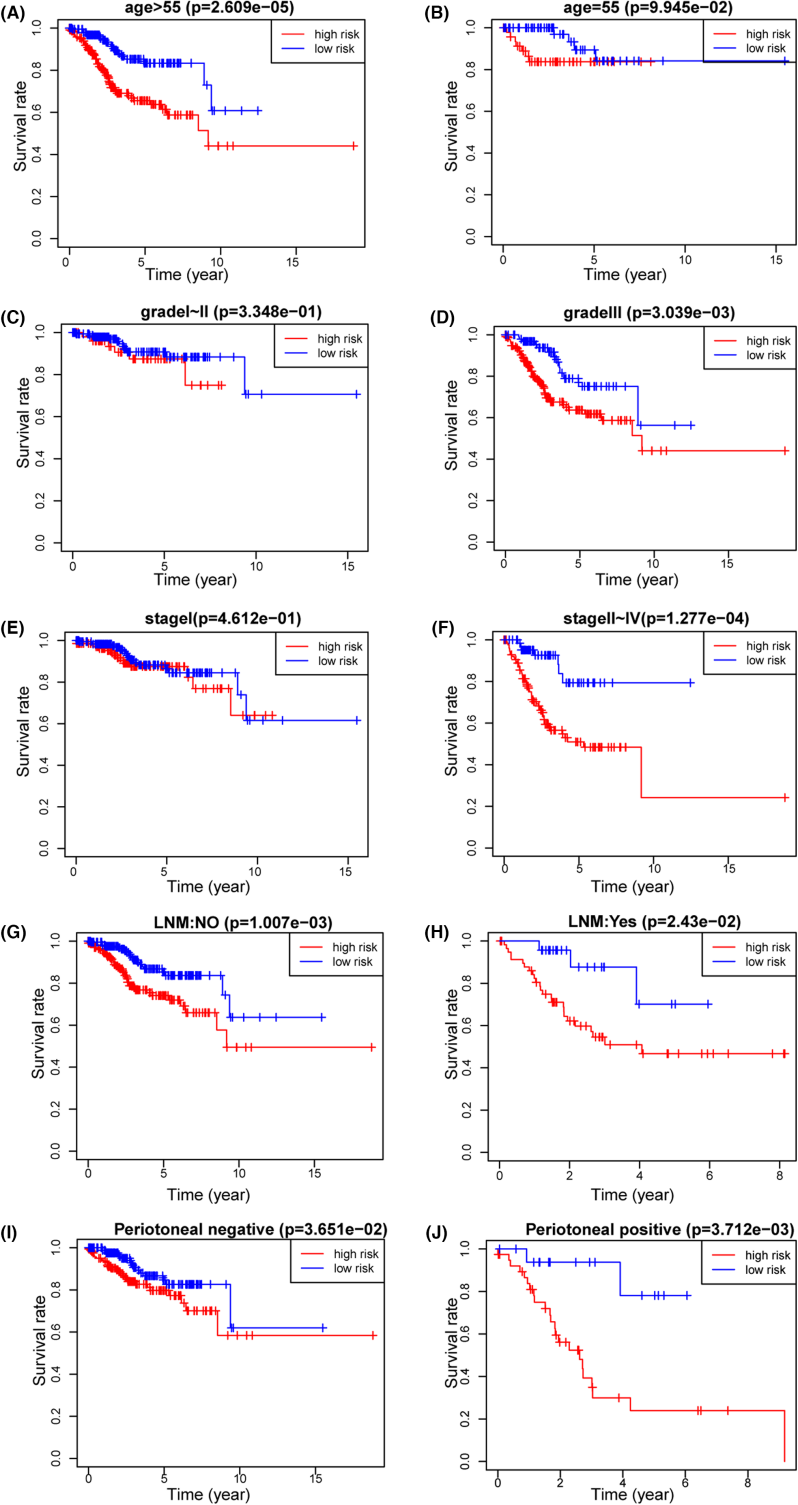
Kaplan–Meier survival by risk groups for patients in the entire cohort and subgroups according to patients' age, stage, grade, and peritoneal cytology. (A) Age ≤ 55.(B) Age > 55. (C) Grade I–II. (D) Grade III. (E) Stage I. (F) Stage II–IV. (G) LNM: negative. (H) LNM: positive. (I) Peritoneal cytology: negative. (J) Peritoneal cytology: positive.

To test whether the prognostic model has the independent predictive ability of age, stage, grade, LNM, and peritoneal cytology, we further grouped the patients into different clinicopathologic subgroups. All results showed that this nomogram could not only accurately differentiate patients in the whole groups but also predict the overall survival in different clinicopathologic subgroups. Patients recognized as low risk had longer survival time than high‐risk patients in different subgroups of grade, age, LNM, stage, and peritoneal cytology (Figure 7A–J).

The nomogram was further confirmed in the 24‐patient clinical cohort from PKUPH which was previously published^23^ (Figure S4). Due to the limited sample size, the predictive power of this model in our cohort is not so good. The nomogram still needs to be validated in another larger sample cohort.

### 3.4 Clinicopathologic relevance analysis and interacting network analysis of five key genes

To further uncover the relationship between the five genes and EC, we analyzed the association between the five risk genes and six clinicopathologic features (LNM, stage, peritoneal cytology, grade, tumor state, and survival status). The results suggested that high OTC expression was correlated with positive peritoneal cytology and patients with tumors (*p* < 0.05) (Figure S5A,B). Compared with patients with a lower grade, tumor‐free, ASNS expression was higher in patients with higher grade, tumor burden (*p* < 0.05) (Figure S5C–E). NR1H4 was not statistically relevant to these clinicopathologic features. Notably, PHGDH and ASRGL1 expression were associated with four or more clinicopathologic features (*p* < 0.05) (Figure S5F–O), which indicates that PHGDH and ASRGL1 promote the initiation and development of EC. Several studies reported that loss of ASRGL1 is an independent prognostic factor in endometrial cancer.^24^, ^25^ PHGDH has been reported to be a potential target in many cancers including breast cancer,^26^ colorectal cancer,^27^ and melanoma.^28^ The heterogeneity of PHGDH might promote cancer metastasis.^29^ However, the function of PHGDH in EC is still unknown. Apart from these, the previous risk model^14^ reported by our group focusing on the whole metabolism also identified PHGDH as an important participator. Data from our hospital patients showed that PHGDH was associated with varieties of clinicopathologic factors, including grade, stage, LNM, and myometrial invasion.^14^ All of these illustrate the importance of PHGDH.

**FIGURE 8:**
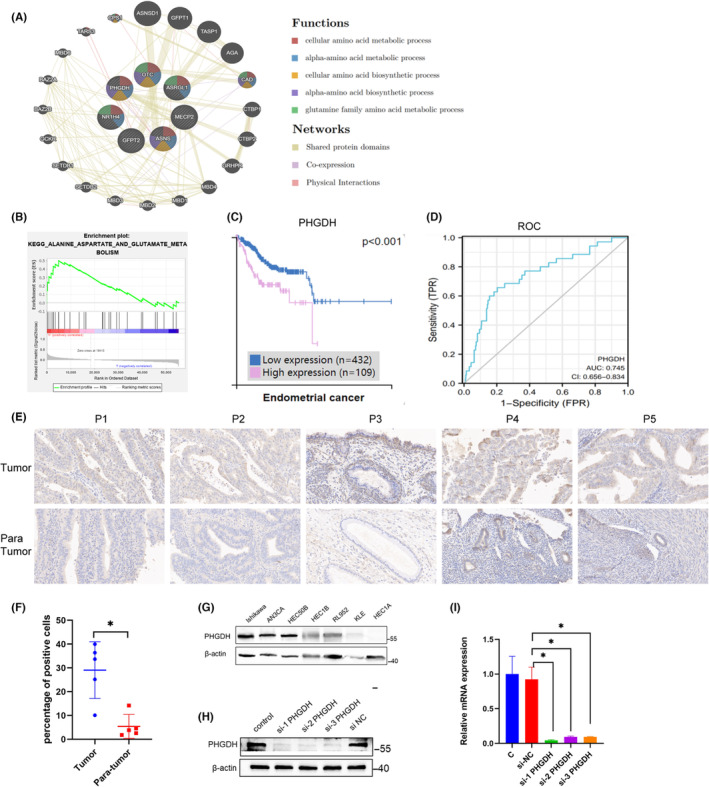
PHGDH is associated with glutamine metabolism and highly expressed in EC. (A) Co‐expression and interacting gene network of five key genes. PHGDH may co‐expressed and physically interacted with ASNS. (B) Single gene enrichment analysis showed positive relationship with PHGDH and aspartate and glutamate metabolism. (C) Survival analysis based on the protein expression of PHGDH from HPA database on EC patients. (D) ROC analysis of PHGDH on EC prognosis prediction. (E) The expression of PHGDH in paired tumor and para‐tumoral tissues from our hospital by IHC and the statistical analysis of the percentage of positive cells (F). (G) The expression of PHGDH in seven endometrial carcinoma cell lines. (H) WB and (I) RT‐PCR was conducted to confirm PHGDH silencing by comparison to the corresponding negative control (NC). *p < 0.05, **p < 0.01.

GeneMANIA analysis was used to figure out the interacted gene network of the five prognostic model genes. The result showed that the gene network points to the glutamine family amino acid metabolic process as well as the amino acid metabolism process (Figure 8A), indicating the intertwined regulation of glutamine and other amino acids. Strikingly, though PHGDH mainly functions in serine metabolism, the enrichment analysis and the interacting network showed that PHGDH was positively correlated to aspartate and glutamate metabolism (Figure 8B), which was closely related to the source and product of glutamine. PHGDH may co‐express and physically interact with ASNS (Figure 8A), a gene that catalyzes glutamine to asparagine. Also, there was a regulatory relationship between the prognostic genes and a variety of cancer‐related genes (Figure 8A). Thus, PHGDH may be a crucial node that connects to serine metabolism and glutamine metabolism, as well as EC progression. Therefore, we decided to explore the role of PHGDH on EC.

### 3.5 PHGDH is highly expressed in EC

Data from HPA database showed that high expression of PHGDH is associated with poor prognosis in EC patients (Figure 8C). ROC curve showed that PHGDH has a good prediction ability with an AUC = 0.745 (Figure 8D). The protein expression of PHGDH was increased in tumor samples compared to the paired para‐tumoral sites in five patients as shown in IHC staining (Figure 8E,F). The clinical information of the five patients was in Table S2. Larger sample validation is needed in future. Next, we compared PHGDH expression in seven EC cell lines. We found that the protein content of PHGDH was higher in the Ishikawa and AN3CA cell lines (Figure 8G). To explore the function of PHGDH on EC cells, we used siRNA‐PHGDH to knockdown PHGDH in Ishikawa and AN3CA cells. Real‐time qPCR and western blot were conducted to examine the knockdown efficiency (Figure 8H,I). As si‐1 PHGDH exhibited the highest knockdown efficiency at the mRNA level, we selected si‐1 PHGDH for subsequent experiments.

### 3.6 Knockdown of PHGDH inhibits cell proliferation, induces apoptosis and represses metastasis of EC cells

**FIGURE 9:**
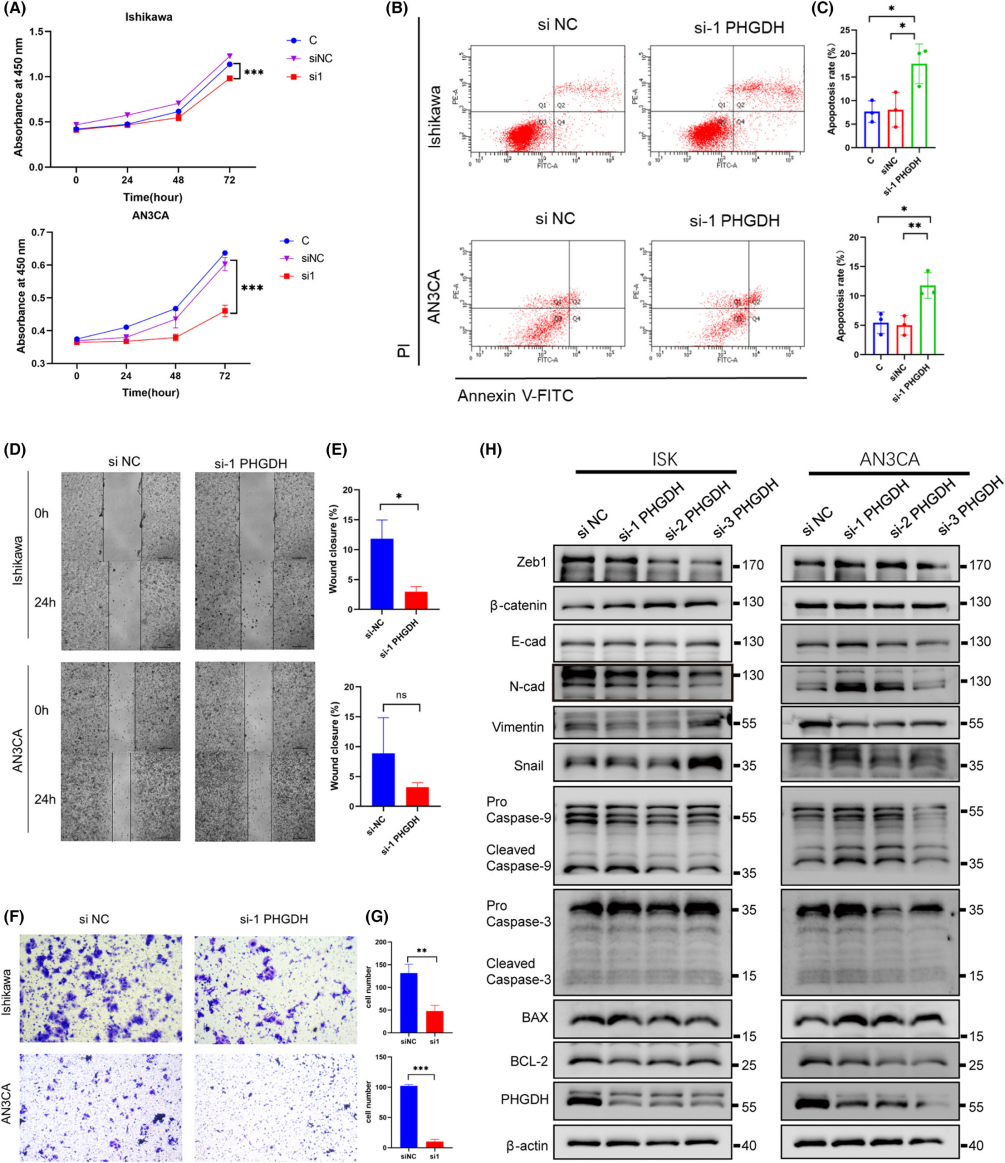
Knockdown of PHGDH inhibits proliferation, induces apoptosis and inhibits metastasis of EC cells. (A) CCK‐8 showed decreased viability on Ishikawa cell and AN3CA cell in response to knockdown of PHGDH. (B, C) Flow cytometry revealed that knocking down of PHGDH induced apoptosis in Ishikawa and AN3CA cells with statistical analysis. Wound healing (D, E) and transwell assays (F, G) were conducted to detect the migration of Ishikawa and AN3CA cells when knocking down PHGDH with corresponding statistical analysis. (H) Western blot of apoptosis markers and EMT markers was performed in si‐PHGDH and si‐NC samples of Ishikawa and AN3CA cell lines. *p < 0.05, **p < 0.01.

CCK‐8 revealed a slight decrease in Ishikawa cell proliferation (*p* < 0.01) and a dramatic decrease in AN3CA cells (*p* < 0.01) in response to PHGDH knockdown (Figure 9A). Flow cytometry showed that the apoptotic cells were increased when knocking down PHGDH in two cell lines (Figure 9B,C). Western blot showed increased caspase‐9, caspase‐3, and Bax, and decreased Bcl‐2 when knocking down PHGDH (Figure 9H). Wound healing and transwell assays were then performed to investigate the effect of PHGDH on the migration of EC cells. The results revealed that PHGDH knockdown significantly inhibited the migration of EC cells in vitro (Figure 9D–G). Western blot showed that when knocking down PHGDH, pro‐EMT transcription factors like Zeb1, and mesenchymal marker, N‐cadherin and vimentin were decreased in ISK cell lines (Figure 9H). Whereas in AN3CA, a cell line derived from lymph node metastatic lesion of endometrial cancer, western blot showed an increase in the epithelial marker E‐cadherin, a decrease in vimentin and Zeb1 (Figure 9H). However, some changes in EMT transcription factors and markers are not consistent with the phenotype, which may be related to cell state, cell line background, migration ability, and different migration mechanisms. Other EMT markers and adhesion molecules should be further employed to elucidate the mechanism.

### 3.7 NCT‐503, a PHGDH inhibitor, inhibits transplanted tumor growth in nude mice

**FIGURE 10:**
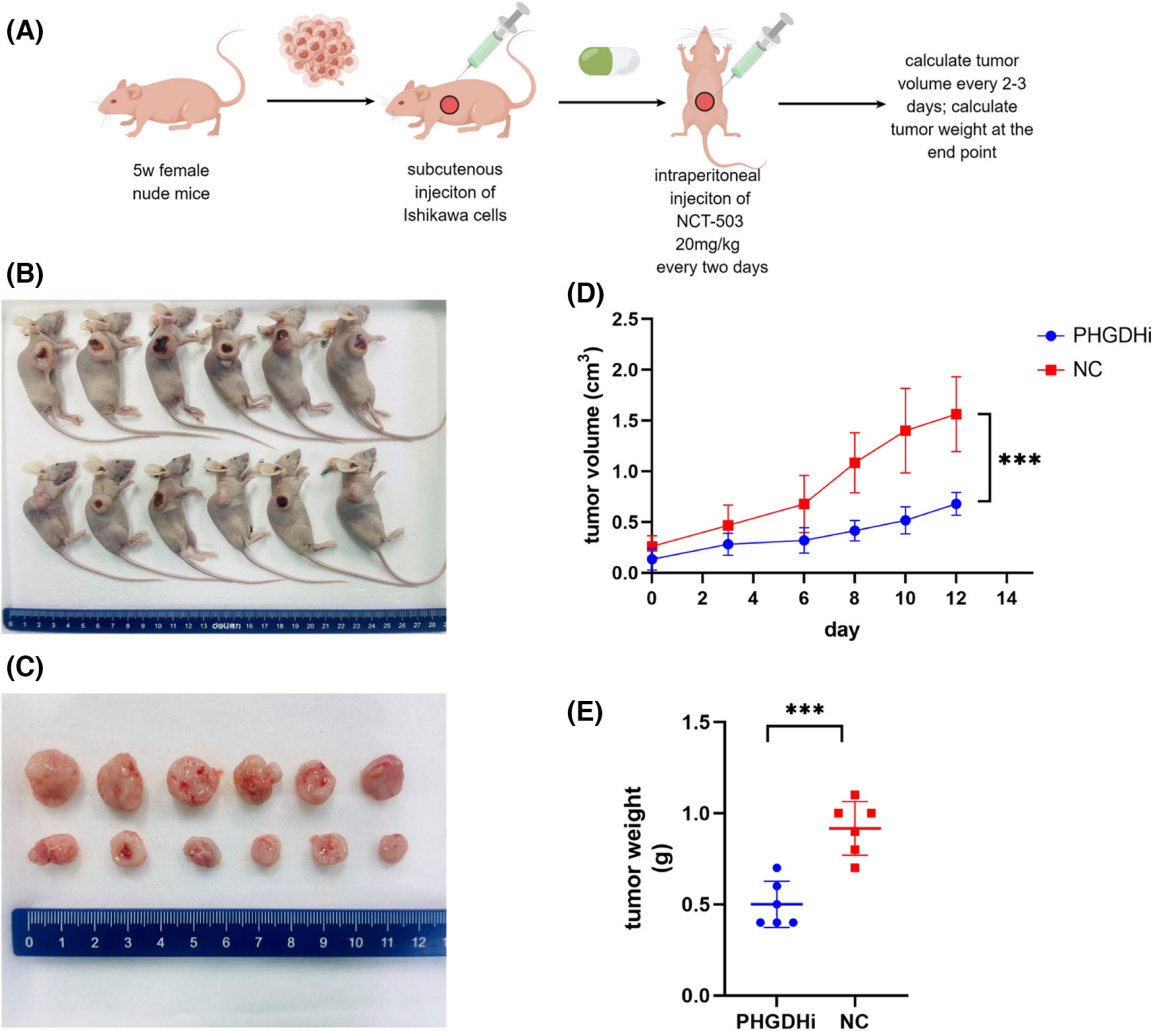
NCT‐503 inhibits endometrial cancer tumor growth in nude mice. (A) Working model of animal procedure generated by FigDraw (http://www.figdraw.com). (B and C) Tumor tissue in situ and excised by surgery at the end of experiment. (D) Tumor volume measured at various times after injection. (E) Weight of two sets of tumors at the end of experiment.*p < 0.05, **p < 0.01.

To further verify the function of PHGDH in vivo, we used Ishikawa cells to construct a subcutaneous tumor‐bearing model in nude mice (Figure 10A). We found that the tumor volume of the NCT‐503^22^ treated group was steadily smaller than that of the control group (*p* = 0.0002) (Figure 10B–D). Additionally, the tumor weight obtained at the endpoint was also significantly slighter than that of the control group, for the average weight of the treated group was 0.50 while the control group was 0.92 (*p* = 0.0004) (Figure 10E). These findings strongly indicated that NCT‐503, an inhibitor of PHGDH, apparently impedes the progression of endometrial cancer.

## 4 DISCUSSION

EC is the most common female genital tract cancer in developed countries and the incidence is increasing annually by 1%–2% as well as disease‐related mortality.^1^ Data showed that the incidence increases by 50% from 1987 to 2008, while the associated death increased by 300%.^1^ Although most patients diagnosed in the early stage have relatively better prognoses, patients with advanced EC‐like recurrent or metastatic disease have poor prognoses with a 5‐year survival of only 17%, and face limited options as chemotherapy and target therapy is lacking efficacy and evidence.^1^ Individual differences and tumor heterogeneity give reasons for treatment resistance or low response. Thus, more accurate and effective tools to evaluate prognosis for giving precise and personalized treatment are needed.

At present, bioinformatics is widely used in biomedical research, including prognosis prediction, diagnostic decision‐making, and molecular typing. It also assists in mechanistic studies including tumor microenvironment and cell ecosystem analysis, tumor heterogeneity evolution, etc. Prognostic prediction tools like nomogram,^13^ a model that takes both clinicopathologic factors and molecular features into consideration, can help stratify patients into different risk groups and give corresponding treatments, which arouse our interest. In the establishment of prognostic models, various regression methods are generally applied to screen variables for the bases of model construction. Common methods for filtering variables include linear regression, polynomial regression, logistic regression, stepwise regression, Cox multivariate regression,^30^ LASSO regression,^31^ Ridge regression, and ElasticNet regression. Cox's proportional hazards regression model, referred to as the Cox regression model, is a semiparametric regression model proposed by British statistician D.R. Cox in 1972. The model can analyze the influence of many factors on survival at the same time based on survival outcome and survival time. It can analyze data with truncated survival time and does not require estimation of the survival distribution type of data. This model is mainly and widely used for the prognostic analysis of tumors and other chronic diseases, and can also be used for etiology exploration in cohort studies. For high‐dimensional and multicollinear data, Lasso, Ridge, and ElasticNet regressions are more suitable.^31^, ^32^ Because when the amount of data is large with many missing values, and the number of independent variables is much larger than the sample size, the traditional Cox regression forward, backward, and stepwise method may not be applicable. In addition to these methods, the random forest, Support Vector Machine, principle component analysis,^33^ deep learning, and extreme gradient boosting of machine learning are also becoming much popular.^32^, ^34^, ^35^ Machine learning are more robust and can outfit imbalanced datasets.^35^ The principal component analysis is also fitted for high‐dimensional data, which can find a smaller set of variables from the original data set, but with the greatest possibility to preserve most of the original information.^33^ However, data mining usually starts with a relatively simple approach. If complex methods do not significantly improve the prediction effectiveness compared to simple methods, researchers tend to choose simple methods. Also, the type, dimension, and complexity of the data also determine the choice of method. In this study, we used a more classical Cox regression‐based nomogram. This method is very common and universal. It is easy to get started and helps researchers solve clinical and scientific questions they care about.

The relationship between EC and metabolic disorders like obesity, diabetes, and hyperlipidemia has been of great interest among worldwide scientists. When BMI increases by 5 units, the incidence of EC increases by 50%.^2^ However, with strong clinical relevance between metabolic factors and EC has been confirmed, the underlying mechanism has not been elucidated yet. Our group has made several efforts to clarify the role of metabolic factors including glucose, insulin, and lipid on EC progression.^4^, ^5^, ^6^, ^36^ Metabolic reprogramming in the tumor is a hallmark of proliferation, metastasis, and tumor signaling. Though glucose was classically thought to be the first choice of tumor utilization as Warburg effect showed, striking research showed that tumor cells use glutamine as an energy resource more preferentially than glucose,^37^ indicating that glutamine metabolism is more significant in cancer development than previously thought. The mechanism of glutamine metabolism in liver cancer, lymphoma, and cervical cancer has been studied. However, studies on the role of glutamine metabolism in EC are limited. Zhou, etc. found that estrogen inhibited autophagy and promoted EC cell growth by upregulated glutamine synthase.^38^ Immunohistochemical analysis in patients with endometrial carcinoma showed that the decrease of Sirt4, which was reported to inhibit glutamine metabolism, was related to the late stage of endometrial carcinoma.^39^ In a large cohort study, obese patients with endometrial and colon cancer were associated with decreased glutamine and increased glutamic acid measured by metabolomics.^40^ However, the role of glutamine metabolism in EC progression is not clear. Thus, we aimed to construct a glutamine metabolism‐based prognostic model and provide potential target genes that may be therapeutic and translational in EC treatment. The model focused on the genes that participated in or interacted with glutamine metabolism regulation, which is more specific than our previous study which focuses on the whole metabolism including glucose, lipid, and amino acids.^14^ Genes identified from the present study may give new insights into the mechanism of how glutamine metabolism participates in EC progression. The interaction between amino acid metabolism and glutamine metabolism as well as cancer‐related pathways are innovative targets for EC cancer therapy. In this study, we first identified five glutamine metabolism‐related genes as prognostic factors in EC. We then built a prognostic nomogram that has good accuracy to predict the EC prognosis. One of the five key genes, PHGDH, which may be an important node linking serine metabolism and glutamine metabolism, was verified in vitro and in vivo, thus may provide a promising target for EC treatment.

We randomly divided the entire TCGA EC cohort into training and testing set ase other public databases like GEO lack survival data for EC patients thus it's not appropriate for validation. By the gene sets retrieved from MSigDB, which involved all genes that participated in glutamine synthesis, catabolism, and regulation, five prognostic‐related and glutamine metabolism‐associated genes, PHGDH, OTC, ASRGL1, ASNS, and NR1H4 were identified by univariate and multivariate Cox regression based on the training cohort and finally participated in the construction of the prognostic model. The predictive efficacy was further validated in the testing and the entire cohort. As indicated by KEGG and GO pathway and literature review, these genes mainly function in different amino acid processes, and partially intervene with glutamine metabolism. The interact gene network analysis by GeneMANIA indicated to the glutamine metabolic process and the amino acid metabolism process, indicating the intertwined regulation of glutamine and other amino acids in cancer progression. Also, the network revealed the correlation of these metabolism‐related genes with cancer signaling genes, indicating their mechanism in tumor progression. PHGDH is the rate‐limiting enzyme in the first step of serine biosynthesis pathway and is involved in the one‐carbon metabolism that is closely related to nucleotide synthesis, DNA methylation, as well as redox hemostasis.^28^ Our analysis showed that PHGDH is positively associated with aspartate and glutamate metabolism, which is the raw material and product of glutamine. Also, PHGDH may co‐express and physically interact with ASNS, which catalyzes the synthesis of asparagine from glutamine. Further, the suppression of PHGDH inhibits the conversion of glutamine to alpha‐KG.^41^ These results indicated that PHGDH might be an interesting and important linker between glutamine and serine metabolism. Studies have demonstrated that PHGDH is upregulated in a variety of cancers, like colorectal cancer, breast cancer, and gastric cancer, regarding cancer initiation, proliferation, differentiation, and metastases.^42^ Elevated PHGDH is associated with poor clinical outcome and pathologic features.^27^ In our study, PHGDH is significantly upregulated in EC and related to unfavorable prognosis. Our previous risk model focusing on the whole metabolism also screened out PHGDH as an important participator,^14^ which further proves the importance of PHGDH in EC prognosis prediction and EC progression. OTC catalyzes the second step of the urea cycle, which synthesizes ornithine to citrulline. Citrulline is a major component for nitrogen and amino acid homeostasis, especially L‐glutamine.^43^ The low expression of OTC was related to larger tumor size and advanced stage, as well as shorter survival in liver cancer.^44^ Also, tumor suppressor P53 may downregulate OTC to inhibit the elimination of ammonia and ureagenesis.^45^ ASRGL, an enzyme involves in the production of L‐aspartate, which may compete with another source of aspartate, the transamination process from glutamine to glutamate. ASRGL, has been reported to promote cell proliferation and suppress apoptosis in various tumors, including breast, ovarian, cervical, and EC.^46^ In EC, it is shown to be a reliable negative prognostic biomarker that is related to overall survival, lymph‐node metastasis, and aggressive clinicopathologic features.^24^, ^25^ Interestingly, studies indicate the relationship of ASRGL1 expression with hormone receptor status,^46^ which builds a link between glutamine and amino acid metabolism with hormone homeostasis, as EC is hormone‐dependent tumor. ASNS catalyzes the synthesis of asparagine from aspartate and glutamine, is overexpressed in several cancers including gastric cancer, liver cancer, breast cancer, and colorectal cancer and promotes cell proliferation, chemoresistance, and metastasis.^47^ NR1H4, a bile acid‐activated nuclear receptor, induces the expression of N‐acetylglutamate synthase to regulate glutamine and glutamate metabolism in liver,^48^ has been implicated in the development of colorectal cancer.^49^

The predictive efficacy of the prognostic model was confirmed by the Kaplan–Meier survival and ROC curve in either the training cohort or testing cohort from the TCGA database and further validated in our hospital's cohort; however, it needed to be validated in a larger clinical cohort. High‐risk groups defined by the prognostic model reveal significantly worse survival compared with the low‐risk group. Also, the prognostic model exhibited good prediction ability for 1‐, 3‐, and 5‐year survival in the ROC curve. The results suggested that the glutamine metabolism‐related prognostic model had a good predictive ability to evaluate the survival of EC patients. In addition, the relevance analysis between the prognostic model and clinicopathologic features showed that a higher risk score was strongly associated with lymph node metastases, positive ascites, and higher tumor stage and grade.

To further elucidate of the function and mechanism of this prognostic model in EC, we performed GSEA enrichment analysis. GSEA analysis identified abnormal “cell cycle, homologous recombination, DNA replication, mismatch repair, insulin signaling, and MAPK pathway” in higher risk groups. These pathways indicate that DNA replication and repair may be the key points that linked glutamine metabolism, amino acid metabolism and EC progression. Glutamine is essential for nucleotide synthesis. Ongoing nucleotide synthesis may cause a load of ROS and frequent donor transfer, which may result in DNA repair and remodeling dysfunction. Glutamine synthase promotes radiation resistance by facilitating nucleotide generation and DNA repair.^50^ NRF2, the transcription factor that mainly response to ROS by influencing levels of NADPH and GSH, was shown to upregulate PHGDH via ATF4.^51^ BRCA1, a DNA repair protein, can interact with NRF2 and promote its stability in response to oxidative stress.^52^ It provides a link for DNA repair, ROS, and amino acid metabolism. For EC, DNA damage repair has been of interest for target therapy and molecular classification. Mismatch repair deficiency (MMRd) is prevalent in EC for almost one‐third of EC tumors,^53^ which may result in a high load of neoantigens that makes immune checkpoint inhibitors more sensible. Clinical trials have recommended anti‐PD‐1 or anti‐PD‐L1 for MMRd EC patients.^53^ Homologous recombination deficiency (HRD) was reported to occur frequently in advanced and recurrent EC and non‐endometroid EC,^54^, ^55^ which is consistent with our results. For patients with HRD, PARP inhibitor may be a choice of target therapy. The interaction of glutamine metabolism and EC in DNA replication and repair may be of a novel promising target for the treatment of high‐risk EC patients. In addition, insulin signaling and MAPK pathways have been reported to be related to metabolic disorders and obesity‐related cancers,^56^, ^57^ especially EC. The insulin signal is known to activate the PI3k‐Akt–mTOR pathway to induce cell proliferation.^56^ Studies have shown that glutamine can increase insulin sensitivity, activate insulin signaling like PI3K‐Akt, and restore glucose homeostasis.^58^, ^59^ MAPK pathway response to growth factors and cellular stress, which promotes EC through immune dysregulation, inflammation, and ER stress. Whereas glutamine deprivation can activate MAPK‐ERK1/2‐p‐DRP1 to promote mitochondrial fragmentation and enhances stemness.^60^ These findings suggest that cancer signaling pathways may participate in the mechanism that glutamine metabolism promotes EC progression, which provides a capable target for EC treatment.

In addition to cancer signaling pathways that may be potential targets for different risk groups, the immune environment analysis indicated that patients evaluated as high risk are related to lower immune score, stromal score and estimation score. Thus, for patients in the high‐risk group, immune therapy may need more consideration. Glutamine is an indispensable nutrient for typical immune responses such as lymphocyte proliferation, cytokine secretion as well as macrophage activation.^61^ The preference uptake of glutamine by cancer cells in the tumor microenvironment may impair the function of the immune system,^9^ which may be the reason that patients in the high‐risk group identified by the glutamine metabolism‐based prognostic model may have an inferior response to immune therapy.

Univariate and multivariate analyses demonstrated that the established risk score, age, and peritoneal cytology were independent prognostic factors in the training cohort and meeting the PHA test. Thus, these three factors were used to generate a nomogram, which can visually display the prognosis of the patients thus facilitating the clinical use. The nomogram owns the advantage of integrating clinical information and molecular features together, for previous studies focus either on genomic biomarkers or clinical characteristics. Our study takes clinicopathologic profiles, molecular aspects, and metabolic mechanisms all into consideration to build a more convincing prognostic model for EC patients. The predicted risk was close to the actual risk demonstrated by the calibration curve. The results confirmed the ability of our model to predict the prognosis and thus give précised medical treatment for EC patients.

Glutamine metabolism not only participates in cancer growth and energy supply, but also interacts with cancer signaling pathways, which provides a novel target for tumor therapy. To further explore the relationship between the five genes in the prognostic model and endometrial carcinoma, we compared variables in the prognostic model with clinicopathologic features. The expression levels of PHGDH and ASRGL1 were significantly correlated with four or more clinicopathologic features (stage, grade, LNM, peritoneal cytology, tumor state, and survival status). Therefore, these two genes may be involved in the progression of EC. Studies reported that low expression of ASRGL1 is an independent prognostic factor in EC.^24^ PHGDH has been reported to be involved in the progression of various cancers and associated with poor prognosis.^42^ However, the influence of PHGDH on the progression of endometrial carcinoma remains unclear. The previous report^14^ by our group focusing on the whole metabolism also identified PHGDH as an important participator, which further indicating its importance in EC prognostic prediction and cancer progression. Data from our hospital showed that PHGDH was associated with unfavorable clinicopathologic factors.^14^ Interestingly, though PHGDH mainly functions in serine metabolism, the enrichment analysis and the interacting network showed that PHGDH was positively correlated to aspartate and glutamate metabolism, which was closely related to the source and product of glutamine. PHGDH may co‐express and physically interact with ASNS, a gene that catalyzes glutamine to asparagine, and other cancer‐related genes. Thus, PHGDH may be a crucial node that connects to serine metabolism, glutamine metabolism, as well as EC progression. Therefore, we further explored the molecular function of PHGDH in EC Ishikawa and AN3CA cells. Our results indicated that the expression of PHGDH was increased in EC tissues and that silencing the expression of PHGDH inhibited cell proliferation, induced apoptosis, and suppressed metastasis in EC. The mechanism may be the induction of pro‐apoptotic proteins like Bax, caspase‐3, and caspase‐9. Also, western blot revealed that pro‐EMT transcription factors like Zeb1 were decreased, as well as the mesenchymal marker, N‐cadherin and vimentin, whereas epithelial marker E‐cadherin was increased, indicating the involvement of these proteins in metastasis mechanism. However, some changes in EMT transcription factors like Snail and beta‐catenin are not consistent with the phenotype experiment, which may be related to cell state, cell line background, migration ability, and different migration mechanisms. Other EMT markers, adhesion molecules, and invasion markers should be further employed to elucidate the mechanism. In addition, PHGDH inhibitor, NCT‐503, was shown to impede tumor growth in vivo effectively. Therefore, PHGDH might represent a promising therapeutic target for the treatment of EC patients, and further investigation is needed.

## 5 LIMITS

The study constructed and validated the prognosis model based on the TCGA database, the most comprehensive tumor database, due to the lack of prognostic information on EC patients in other public databases. The prognosis model was verified on the small size cohort of our hospital, but a larger sample size and multicenter samples are needed to further evaluate and verify the clinical application of this prognosis model.

In this study, we verified the function of PHGDH in vivo and in vitro in the progression of EC and proposed that PHGDH as an enzyme linking glutamine and serine metabolism may be a new target for the treatment of EC. However, a more in‐depth mechanism study is needed in the future and further biochemical analysis of glutamine metabolites when blocking PHGDH is needed.

This study constructed and verified the prognosis model of EC based on glutamine metabolism for the first time, and further exploration is needed to compare the prediction accuracy of this prognostic model with other molecular prognosis models in the future, to help clinicians make better clinical decisions. Also, the study mainly used Cox regression‐based nomogram as the bioinformatic approach, other innovative and advanced bioinformatic means like machine learning should be applicated in the future to compare the results between different methods.

## 6 CONCLUSION

This was the first study that established a prognostic model in EC patients based on the profile of glutamine metabolism‐related genes. We used a public database to construct and validate its accuracy. DNA replication and repair abnormality identified by pathway enrichment analysis may be the crucial point that linked glutamine metabolism and EC progression. Also, patients in higher risk stratified by the model may represent a lower response to immune therapy. The function and aggressive phenotype of PHGDH were explored in endometrial cell lines and xenograft mice models. Therefore, the prognostic model and nomogram are favorable tools to group patients and provide personalized treatment to improve the prognosis of the patients.

## AUTHOR CONTRIBUTIONS

**Lirong Zhai:** Data curation (lead); formal analysis (lead); investigation (equal); methodology (equal); writing – original draft (lead). **Xiao Yang:** Data curation (equal); formal analysis (equal); funding acquisition (supporting); methodology (equal); software (equal); writing – review and editing (supporting). **Yuan Cheng:** Funding acquisition (supporting); supervision (equal); writing – review and editing (supporting). **Jianliu Wang:** Conceptualization (equal); funding acquisition (lead); project administration (lead); resources (lead); supervision (lead); writing – review and editing (supporting).

## FUNDING INFORMATION

This work was supported by National Natural Science Foundation Key Program (82230050), the National Natural Science Foundation General Program (82072861, 82173119, 82203568), Peking University Medicine Fund of Fostering Young Scholars' Scientific & Technological Innovation (BMU2022PYB028), Project (RDJP2022‐09) supported by Peking University People's Hospital Research and Development Funds.

## CONFLICT OF INTEREST STATEMENT

The authors declare that the research was conducted in the absence of any commercial or financial relationships that could be construed as a potential conflict of interest.

## ETHICS APPROVAL AND CONSENT TO PARTICIPATE

The study was approved by the Ethics Committee of Peking University People's Hospital (2020PHB424‐01). Any patient information included in this article has been consented for publication. All animal care and procedures were in accordance with national and institutional policies for animal health and well‐being and approved by the Laboratory Animal Ethics Committee of Peking University People's Hospital (Ethics approval number: 2020PHE094).

## CONSENT FOR PUBLICATION

Informed consent was obtained from all subjects involved in the study.

## Supporting information


Figure S1.
Figure S2.
Figure S3.
Figure S4.
Figure S5.
Table S1.
Table S2.

## Data Availability

All data generated or analyzed during this study are included in this published article and its supplementary information files.
