# Supplementary material for: Glutamine and amino acid metabolism as a prognostic signature and therapeutic target in endometrial cancer
Source: Cancer Med. 2023 Jun 30;12(15):16337–58. doi: 10.1002/cam4.6256 (PMC10469729; doi:10.1002/cam4.6256)
Supplement: Supplementary file 1 — Figure S1. Figure S2. Figure S3. Figure S4. Figure S5. Table S1. Table S2. [file CAM4-12-16337-s001.docx]

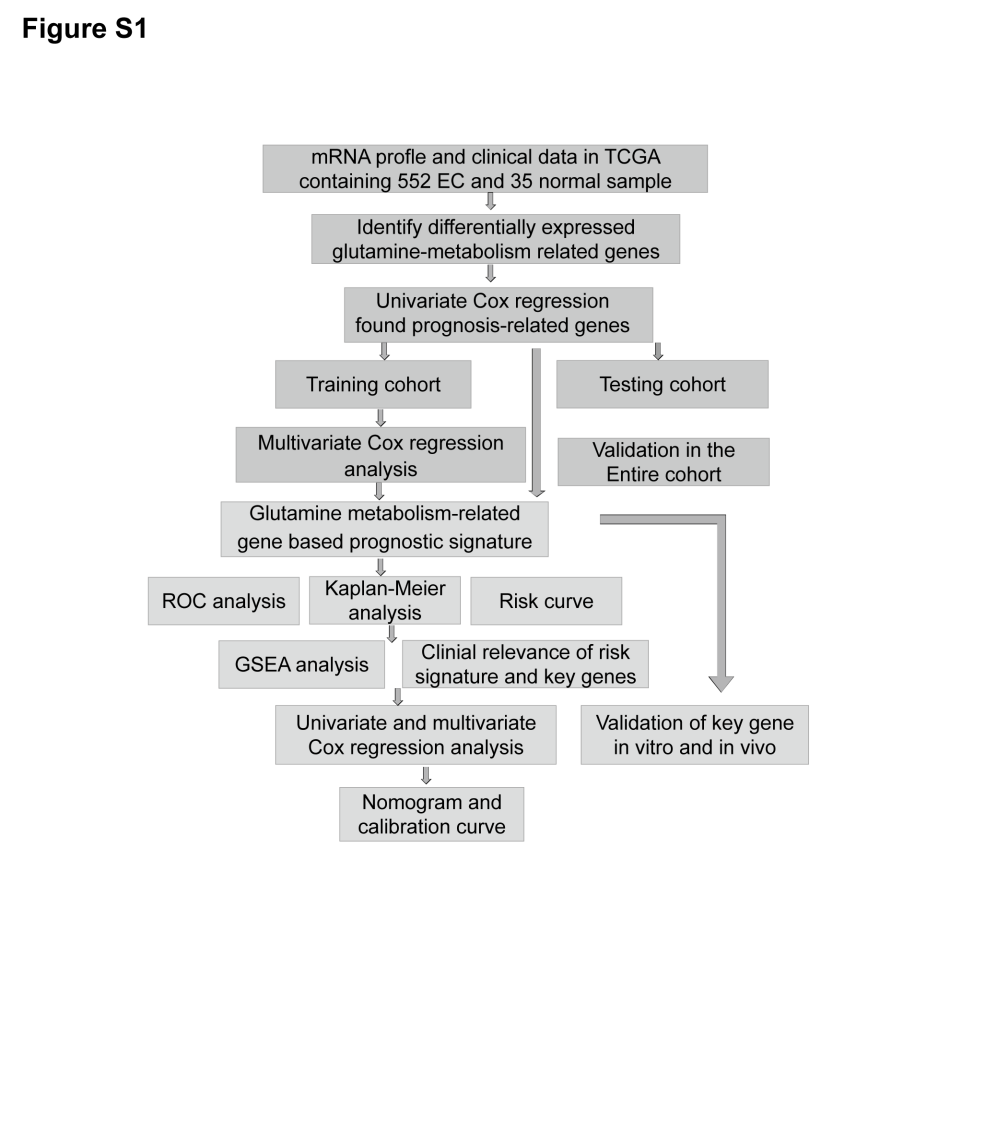


**Figure S1.** The flow diagraph for establishing glutamine metabolism-related prognostic model in EC.


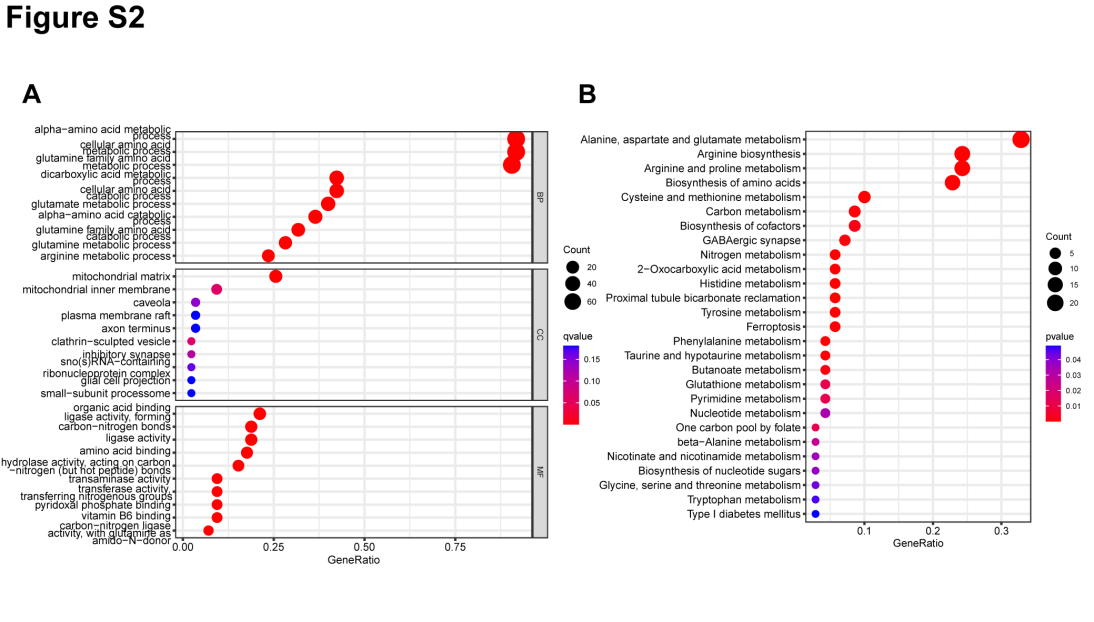


**Figure S2. E**nrichment analysis of all glutamine metabolism-related genes. Go(A)and KEGG(B) enrichment analysis. X-axis represents gene ratio, and different color represents -log10 (FDR).

**Table S1** List of differently expressed glutamine metabolism-related genes in EC (FDR < 0.05, ｜logFC｜ > 1).

| **Gene** | **conMean** | **treatMean** | **logFC** | **pValue** | **fdr** |
| --- | --- | --- | --- | --- | --- |
| KLA | 0.141942 | 1.127752 | 2.990076 | 2.59E-14 | 1.79E-13 |
| GAD1 | 0.228738 | 4.406531 | 4.267878 | 4.20E-11 | 1.81E-10 |
| ADHFE1 | 2.724378 | 0.714015 | -1.9319 | 1.29E-17 | 1.64E-16 |
| PHGDH | 13.44894 | 30.01747 | 1.15831 | 2.28E-08 | 6.92E-08 |
| LGSN | 0.03021 | 0.51864 | 4.101635 | 4.10E-05 | 7.42E-05 |
| PYCR1 | 6.244155 | 32.29994 | 2.370953 | 1.40E-18 | 3.74E-17 |
| ALDH18A1 | 20.06677 | 50.44876 | 1.330011 | 1.97E-18 | 3.74E-17 |
| GCLC | 11.25208 | 5.505778 | -1.03117 | 0.00908 | 0.012324 |
| ART4 | 0.272207 | 0.027215 | -3.32222 | 5.19E-16 | 4.93E-15 |
| SIRT7 | 2.64779 | 5.445997 | 1.040407 | 3.32E-16 | 3.61E-15 |
| MECP2 | 6.118123 | 2.987727 | -1.03404 | 1.40E-19 | 1.07E-17 |
| GGT1 | 1.440008 | 7.73419 | 2.425173 | 4.06E-13 | 2.06E-12 |
| OTC | 0.696783 | 0.071424 | -3.28623 | 7.11E-12 | 3.38E-11 |
| ASRGL1 | 19.46317 | 53.00201 | 1.4453 | 6.70E-07 | 1.54E-06 |
| ASNS | 5.269782 | 10.71224 | 1.023445 | 2.36E-09 | 7.80E-09 |
| PRODH | 0.103215 | 0.718998 | 2.800331 | 0.015876 | 0.021168 |
| PYCR3 | 5.022857 | 12.38102 | 1.30155 | 5.03E-14 | 2.94E-13 |
| AGMAT | 0.651499 | 3.18299 | 2.288548 | 3.88E-18 | 5.90E-17 |
| GLYATL1 | 0.120965 | 0.337597 | 1.48071 | 0.001738 | 0.002642 |
| ATP2B4 | 62.86667 | 23.99958 | -1.38929 | 2.77E-13 | 1.51E-12 |
| GFPT2 | 5.251759 | 1.628389 | -1.68936 | 2.02E-15 | 1.71E-14 |
| GOT1 | 10.91149 | 22.56226 | 1.048063 | 1.95E-18 | 3.74E-17 |
| NR1H4 | 0.387513 | 0.127041 | -1.60895 | 0.000147 | 0.000242 |


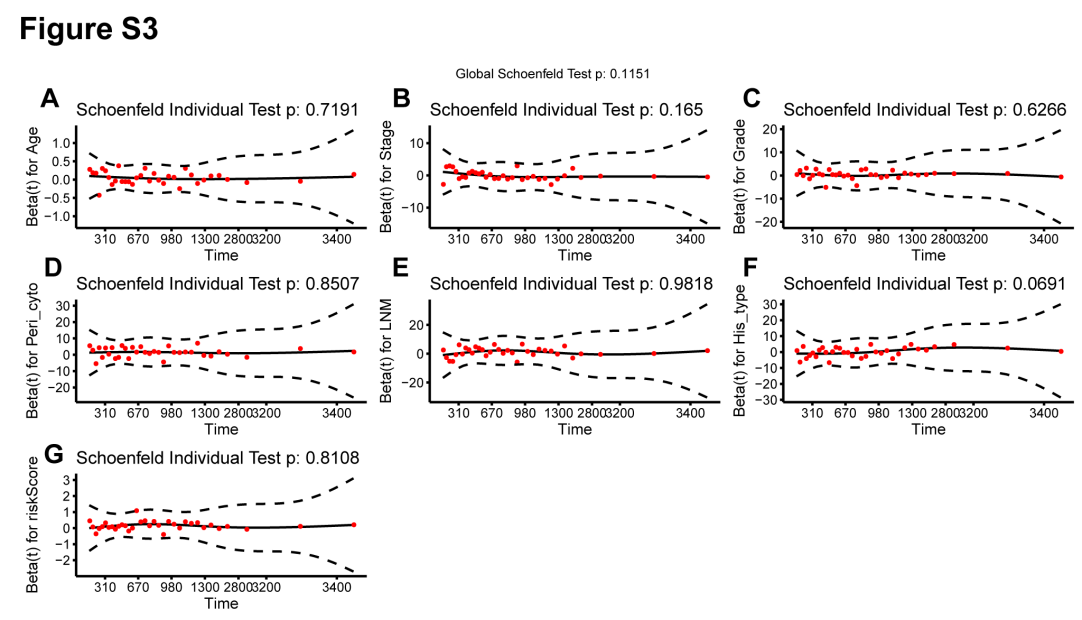


**Figure S3.** Proportional hazard assumption test of all variables. The horizontal axis represents time, and the vertical axis is beta values for each variable. Red dots are the residuals of beta values of different variables. Black solid line is the fitting curve of all residuals. Dashed lines are the lower and upper limits of the 95% CI of all residuals


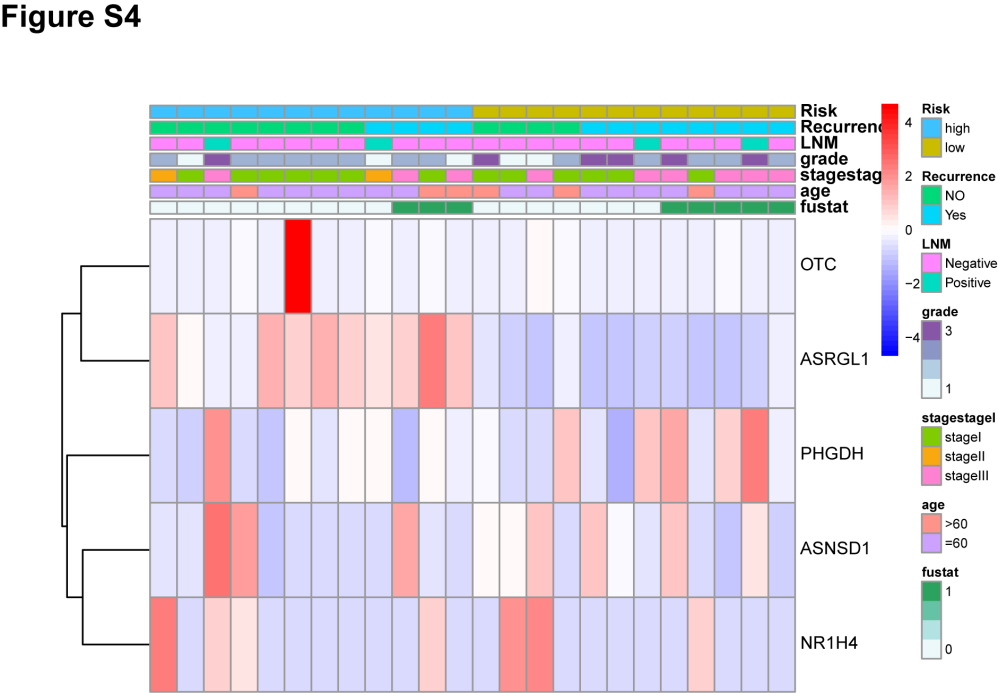


**Figure S4.** Validation of the Nomogram Based on the PKUPH clinical Samples. Heatmap of clinicopathological factors and the five hub genes in the high-risk and the low-risk groups.


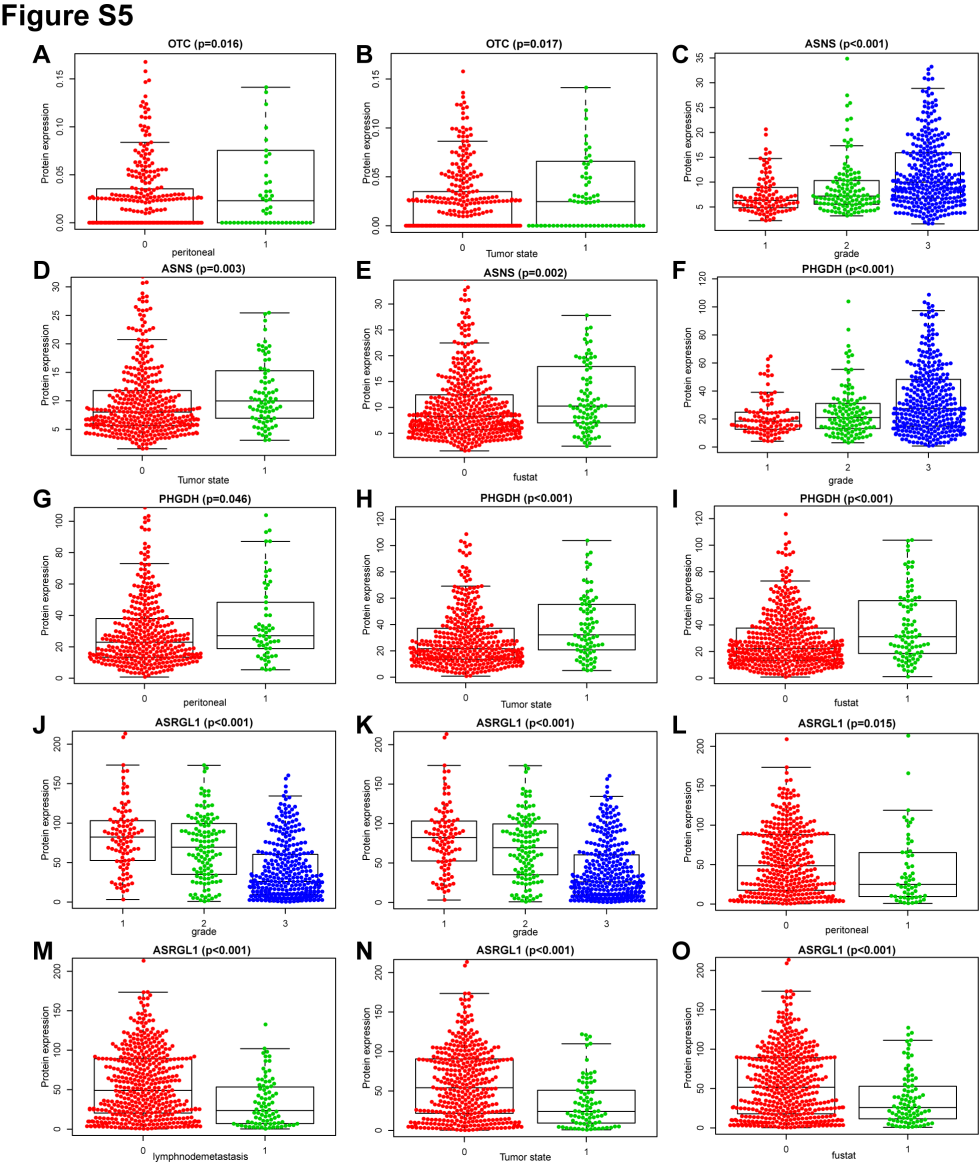


**Figure S5.** The relevance of the expression of OTC, ASNS, PHGDH, and ASRGL1 and clinicopathologic factors including stage, grade, LNM, peritoneal cytology, tumor state and survival status. Only p value of the relevance lower than 0.05 was shown.

**Table S2** The clinic information of IHC tissues from PKUPH

| ID | tissue | age | grade | stage | LVSI | MI | LNM | CI | SOM |
| --- | --- | --- | --- | --- | --- | --- | --- | --- | --- |
| P1 | EC | 65 | G3 | IIIC1 | positive | >1/2 | positive | negative | negative |
| P2 | EC | 59 | G2 | IB | positive | >1/2 | negative | negative | negative |
| P3 | EC | 70 | G2 | IA | negative | <1/2 | negative | negative | negative |
| P4 | EC | 50 | G2 | IB | positive | <1/2 | negative | negative | negative |
| P5 | EC | 58 | G3 | IIIA | negative | >1/2 | negative | negative | negative |

LVSI: lymph vascular involvement; MI: myometrial invasion; LNM: lymph node metastasis; CI: cervix stromal involvement; SOM: salpingo-ovarian metastasis.
